# Molecular mechanism of exchange coupling in CLC chloride/proton antiporters

**DOI:** 10.1101/2025.05.08.652968

**Published:** 2025-05-09

**Authors:** Deniz Aydin, Chih-Ta Chien, Jürgen Kreiter, Amy Nava, Jasmina M. Portasikova, Lukas Fojtik, Catalina Mosquera Salcedo, Petr Man, Ron O. Dror, Wah Chiu, Merritt Maduke

**Affiliations:** 1 Stanford University, Department of Molecular and Cellular Physiology; 2 Department of Computer Science, Stanford University, Stanford, CA 94305; 3 Department of Structural Biology, Stanford University, Stanford, CA 94305; 4 Institute for Computational and Mathematical Engineering, Stanford University, Stanford, CA 94305; 5 Department of Bioengineering and Department of Microbiology and Immunology, Stanford University, Stanford, 94305; 6 National Institute of Diabetes and Digestive and Kidney Diseases (NIDDK), National Institutes of Health, Bethesda, MD, 20892; 7 Department of Biochemistry, Faculty of Science, Charles University, Hlavova 2030/8, 128 43, Prague 2, Czech Republic; 8 Institute of Microbiology of the Czech Academy of Sciences, Division BioCeV, Prumyslova 595, 252 50 Vestec, Czech Republic; 9 Division of CryoEM and Bioimaging, SSRL, SLAC National Accelerator Laboratory, Stanford University, Menlo Park 94025

## Abstract

The ubiquitous CLC membrane transporters are unique in their ability to exchange anions for cations. Despite extensive study, there is no mechanistic model that fully explains their 2:1 Cl^‒^/H^+^ stoichiometric exchange mechanism. Here, we provide such a model. Using differential hydrogen-deuterium exchange mass spectrometry, cryo-EM structure determination, and molecular dynamics simulations, we uncovered new conformational dynamics in CLC-ec1, a bacterial CLC homolog that has served as a paradigm for this family of transporters. Simulations based on a cryo-EM structure at pH 3 revealed critical steps in the transport mechanism, including release of Cl^‒^ ions to the extracellular side, opening of the inner gate, and novel water wires that facilitate H^+^ transport. Surprisingly, these water wires occurred independently of Cl^‒^ binding, prompting us to reassess the relationship between Cl^‒^ binding and Cl^‒^/H^+^ coupling. Using isothermal titration calorimetry and quantitative flux assays on mutants with reduced Cl^‒^ binding affinity, we conclude that, while Cl^‒^ binding is necessary for coupling, even weak binding can support Cl^‒^/H^+^ coupling. By integrating our findings with existing literature, we establish a complete and efficient CLC 2:1 Cl^‒^/H^+^ exchange mechanism.

The CLC “chloride channels” are a biophysically fascinating gene family consisting of both passive chloride (Cl^‒^) channels and active transporters that exchange chloride for protons (H^+^)^1–5^. CLC transporters are ubiquitously expressed in intracellular membranes, and their dysfunction disrupts ion homeostasis and causes Dent’s disease, osteopetrosis, and neurodegenerative disorders^4,6–12^. CLC transporters are also found widely in plants and microorganisms^4,13,14^. In bacteria, CLC transporters support pathogenesis by facilitating extreme acid tolerance^15,16^.

CLC transporters catalyze Cl^‒^/H^+^ exchange transport with a stoichiometry of 2 Cl^‒^ ions per H^+^. This 2:1 stoichiometry is observed in all CLC transporter homologs under all experimental conditions measured – including Cl^‒^ and H^+^ concentrations that vary by orders of magnitude. CLC-ec1 is an *E. coli* homolog that was initially examined due to its functional similarity to mammalian homologs, together with its superior suitability for structural analysis^17–19^. CLC-ec1 was the first CLC demonstrated to be an active transporter instead of a channel^1^, and has proven valuable for guiding studies of mammalian CLCs, both transporters and channels^2,3,20–22^. CLC-ec1 remains a useful paradigm, as it is currently the only transporter homolog for which multiple conformations have been determined at high resolution^19,23–25^ and the only homolog whose ion-binding properties have been quantified by isothermal titration calorimetry^26,27^. In addition, the availability of a functional cysteineless variant makes it amenable to spectroscopic approaches^28–30^.

Studies of CLC-ec1’s structure, function, and dynamics have provided essential clues to the CLC Cl^‒^/H^+^ transport mechanism. Structurally, CLCs are homodimers in which each subunit can act independently^4^. Each subunit contains permeation pathways for Cl^‒^ and H^+^, which are shared along the extracellular section and then diverge toward the intracellular side (Fig. 1a). The Cl^‒^ pathway is gated by a conserved glutamate (“E_gate_” – E148 in CLC-ec1), which also functions as a H^+^-transfer site (Fig. 1a). This pathway is defined by two anion-binding sites, S_ext_ and S_cen_, in the middle of each subunit. These sites can be occupied by the negatively charged E_gate_ side chain (“middle” and “down” conformations in Fig.1b) or by Cl^‒^ ions. The H^+^ permeation pathway diverges from the Cl^‒^ pathway toward the intracellular side, as illustrated in Fig.1a. This positioning of the H^+^ pathway is supported by several lines of evidence. First, mutations along the intracellular segment of the pathway, particularly at “Glu_in_” (E203), severely repress H^+^ transport^31,32^. Second, E_gate_ can occupy this proposed H^+^ pathway in its “out” conformation (see Fig.1b). Third, molecular dynamics simulations on CLC-ec1 detect water wires (chains of hydrogen-bonded water molecules) that provide a pathway for H^+^ conduction in this region^24,25,32–39^. Within this framework, the key to achieving 2:1 Cl^‒^/H^+^ coupling must lie in coupling of the E_gate_ rotameric conformational changes to Cl^‒^ and H^+^ movement in opposite directions through the pathways.

Although some important aspects of this coupling are understood—such as the correlation between coupling and Cl− binding to S_cen_^26,32,37,40,41^ and the synergistic binding of Cl− and H+^26,27^ —a satisfactory model to fully explain the 2:1 exchange mechanism has remained elusive. Existing models explain some of CLC-ec1’s known properties but fall short of capturing the full picture. For instance, proposed models tend to be coherent in only one direction of the transport cycle^24,38,42^, whereas CLC-ec1 facilitates transmembrane Cl^‒^/H^+^ exchange with similar efficiency in both directions^43^. Furthermore, all available structures show the inner gate of the Cl^−^ pathway as closed, leaving the mechanism of Cl^‒^ movement to and from the intracellular side unresolved.

A major challenge in the study of CLC antiporters is the apparently subtle nature of the protein conformational changes, which is in stark contrast to the large conformational changes that are seen in other secondary active transporters^44–46^. For instance, the crystal structure of WT CLC-ec1 shows E_gate_ in the “middle” conformation (Fig.1b). When E_gate_ is mutated to glutamine (to simulate the protonation that occurs as part of the transport cycle), the resulting structure is nearly indistinguishable from that of WT, differing only in the rotation of the side chain (from “middle” to “up”), and with the extracellular pathway still narrower than a Cl^−^ ion. This observation raised an important question: are conformational changes in CLC transporters inherently subtle compared to those in other transporters, or do significant conformational shifts remain undiscovered? This question drove us, and others, to explore these changes outside the constraints of crystallization^30,47–51^. To do so, we utilized low-resolution spectroscopic techniques to analyze conformational dynamics in E_gate_ mutant proteins, as well as in wild-type proteins under conditions of lowered pH to facilitate the protonation of E_gate_. Through these studies, we identified a mutant (“QQQ”) that was stabilized in the protonated state, allowing us to determine its high-resolution structure and reveal molecular details of conformational change, including widening of the extracellular pathway and the positioning of E_gate_ in the “out” conformation^24^. Subsequently, structures of WT CLC-ec1 at pH 4.5 recapitulated many of the conformational features observed in the QQQ structure as well as new conformational changes in the N-terminal region and at the dimer interface^25^. However, none of these structures illuminate the mechanism of inner-gate opening. This void leaves lingering questions and suggests we may still be missing important conformational changes, particularly given conformational dynamics observed spectroscopically in structures lining the inner Cl^−^ pathway^47,50^.

Here, we explored CLC-ec1’s pH-dependent conformational change at even lower pH. CLC-ec1 chloride transport rates increase as the pH is lowered from 4.5 to 3.0^52^, demonstrating the protein’s ability to undergo conformational cycling under these conditions. Additionally, low pH aligns with CLC-ec1’s physiological role in extreme acid tolerance within the mammalian stomach^15^. When we lowered the pH from 4.5 to 3.0, we observed a sharp increase in protein conformational dynamics detected by hydrogen deuterium exchange spectrometry. This observation prompted us to explore the molecular details of this conformational change using single particle cryo-electron microscopy (cryo-EM) structure determination. Our cryo-EM structures revealed subtle but consequential conformational changes between pH 4.0 and 3.0, with molecular dynamics (MD) simulations of the pH 3.0 structure providing unprecedented insights, including new water wires and opening of the inner gate. Integrating results from structure, function, and simulation studies, we present a model to explain the bidirectional 2:1 Cl^‒^/H^+^ stoichiometric exchange mechanism of CLC exchange transporters.

## RESULTS

### Hydrogen-deuterium exchange mass spectrometry reveals two pH-dependent conformational transitions in CLC-ec1

To investigate pH-dependent changes in CLC-ec1 conformational dynamics, we first employed hydrogen-deuterium exchange mass spectrometry (HDX-MS). This technique offers an unbiased (label-free) spectrometric approach to revealing structural properties and conformational dynamics in proteins^53,54^ with virtually no limitation in experimental conditions (pH, temperature, buffer composition). It is especially advantageous for studying CLC-ec1 because it can identify subtle conformational changes in confined areas, such as the inner gate, without requiring a bulky label. HDX is based on the principle that structurally rigid, H-bonded protein regions incorporate deuterium into backbone amides slowly, whereas water-accessible dynamic protein regions do so rapidly.

**Extended Data Figure 1.**
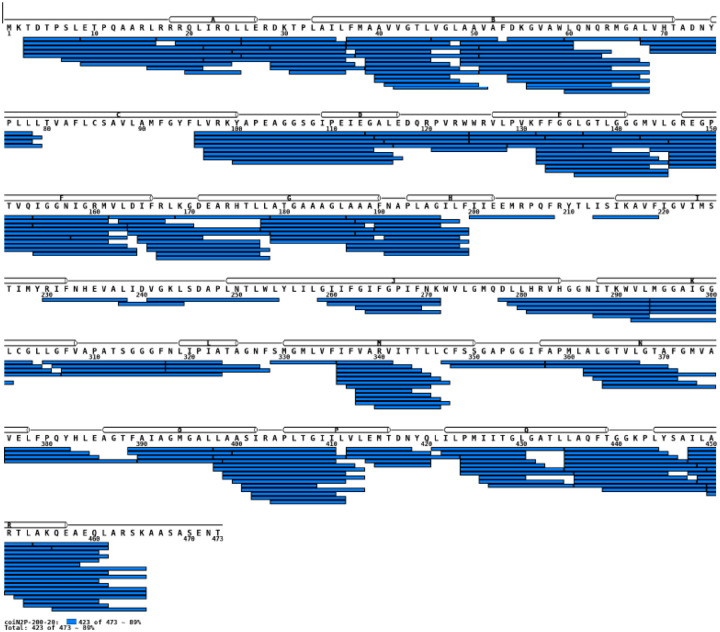
Sequence coverage map of CLC-ec1 by peptides providing HDX data. Online proteolysis of CLC-ec1 using co-immobilized Nepenthesin-2/Pepsin column (bed volume 70 μl). Digestion conditions: 19°C, 0.4% formic acid, flow rate of 200 μL min^−1^. Each peptide is represented by a blue bar. Secondary structure elements are shown above the sequence. Almost complete (89%) sequence coverage was obtained through 237 peptides with average peptide length of 11.4 amino acids and average redundancy of 6.4.

CLC-ec1 was overexpressed in *E. coli* and purified using established methods^19,24,52^. The concentrated protein sample was incubated in deuterated buffer for varying amounts of time to allow hydrogen atoms in the protein backbone to exchange with deuterium from the solvent. After the incubation period, the exchange reaction was quenched to stop further deuterium uptake, and the sample digested with protease. The resulting peptides were analyzed using mass spectrometry (MS), which measures the mass-to-charge ratio of the peptides, allowing for the determination of the extent of deuterium incorporation and rates of hydrogen deuterium exchange (HDX). MS analysis of CLC-ec1 identified 237 peptides, providing nearly complete (89%) coverage of the protein sequence (Extended Data Fig.1).

**Extended Data Figure 2:**
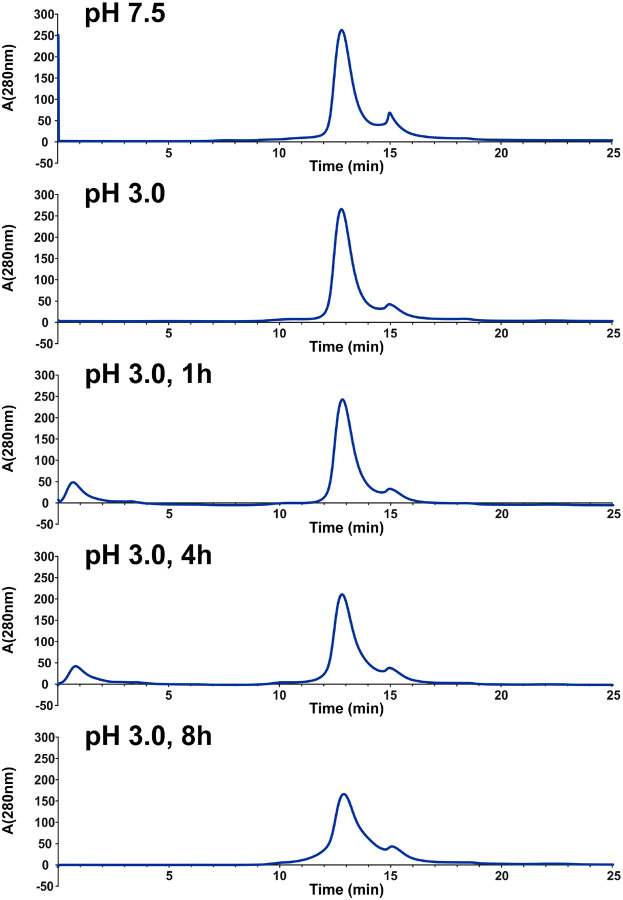
CLC-ec1 stability at pH 3.0. Size exclusion chromatography of CLC-ec1 using EnrichTM SEC 650 10×300 24 mL (Biorad), flow rate 1 mL/min. The top panel shows CLC-ec1 prepared at pH 7.5. The lower panels show CLC-ec1 assessed using size exclusion chromatography immediately after preparation at pH 3.0 and at several time points, demonstrating stability over an 8-hour period.

Fig. 2a shows a differential heat map for deuterium incorporation observed in CLC-ec1 at pH 4.5 compared to pH 6.5. Each row shows comparisons at specific time points where the samples had equivalent opportunity for deuterium exchange, accounting for the intrinsic pH-dependence of the hydrogen deuterium exchange reaction, which is 100-fold faster at pH 6.5 than at pH 4.5. Several regions of the CLC-ec1 protein exhibited increased HDX at pH 4.5, particularly around helices A, G, H, I, P, and R, which are located on the intracellular side of the protein and along the subunit interface (as mapped onto the protein structure at right in Fig.2a). This result closely aligns with previous cryo-EM structural analyses at pH 7.5 and pH 4.5, which showed conformational changes consistent with enhanced water accessibility in these regions at the lower pH^25^. Fig.2b shows a differential heat map for deuterium incorporation observed in CLC-ec1 at pH 3.0 compared to pH 4.0. In this case, many peptides displayed increased HDX at the lower pH. This result indicates a second conformational transition, encompassing a broad swath of the protein. Importantly, the CLC-ec1 protein remained stable under the pH 3.0 condition, as evaluated by size exclusion chromatography (Extended Data Fig.2), indicating that the increase in HDX is not merely a result of protein unfolding.

**Extended Data Figure 3:**
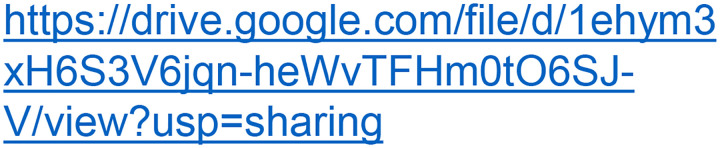
Deuterium uptake plots for all CLC-ec1 peptides providing HDX data. Five experimental conditions were followed: pH 3.0 (black), pH 3.5 (grey), pH 4.0 (blue), pH 4.5 (cyan) and 6.5 (orange). Exchange times are normalized to pH 3.0 to compensate for the difference in the exchange rate. For replicated time points (20s, 633s, and 20000s) average values with respective standard deviations are shown in plots. Data were corrected for back-exchange. Peptide limits and charge states are shown above each plot.

The deuterium uptake plots used to create the HDX heat maps are presented in Extended Data Fig.3. Fig.2c illustrates examples of these uptake plots for peptides selected to represent the HDX properties and conformational transitions observed in this study. The location of each peptide (residue numbers indicated above each plot) was mapped in color onto the protein structure shown at center. For reference, a diagram of the protein with all helices labeled is shown in Fig. 2d. Peptides 336–344 (helix M, dark green) and 41–50 (helix B, pink) (plots shown at top) represent regions of the protein that display little to no HDX at any pH. These peptides are in helices within the hydrophobic core of the protein/membrane region. Peptides 17–25 (helix A, yellow) and 200–208 (helix H, blue) (plots at left) represent the first pH-dependent conformational transition, as detected by the major shift in the deuterium incorporation curve that occurs at pH 4.5 compared to pH 6.5. Peptides 146–163 (helix F, orange) and 110–116 (helix D, teal green) (plots at right) represent the second pH-dependent conformational transition, detected by the shift in the deuterium update plot occurring at pH 3.0 compared to 3.5. Notably, these peptides encompass helices that constitute the outer and inner gates of the Cl^‒^ pathway, where enhanced water accessibility may indicate increased mobility of Cl^‒^ and H^+^ in these regions at pH 3.0. In contrast, water accessibility of other membrane helices (B, I, M, and N) remained low at pH 3.0 (Extended Data Fig.3). Accessibility of helices C and J was also low, although HDX coverage of these helices was not complete. Taken together, these data demonstrate two distinct pH-dependent conformational changes in CLC-ec1, one of which had not been previously detected.

### Cryo-EM structures of CLC-ec1

**Extended Data Fig. 4:**
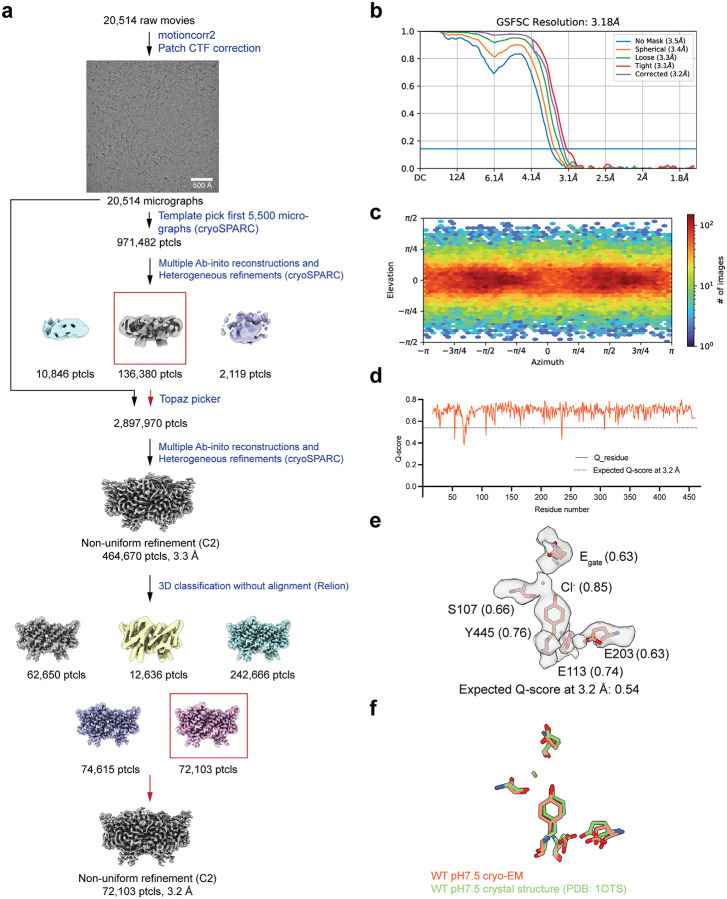
Cryo EM workflow and validation data for CLC-ec1 at pH 7.5. (**a**) Cryo-EM data processing workflow. (**b**) Gold-standard FSC curve. The resolution is estimated based on FSC at 0.143. (**c**) Angular distribution plot (**d**) Per-residue Q-score as a function of residue number. The expected Q-score at the map resolution is indicated by the dotted line. (**e**) The cryo-EM density and molecular model overlay for central Cl^‒^ binding site. The key residues and bound Cl^‒^ and their corresponding Q-score are annotated. (**f**) The overlay of our cryo-EM structure (salmon color) with the previous crystal structure (green) (PDB: 1OTS).

**Extended Data Fig. 5.**
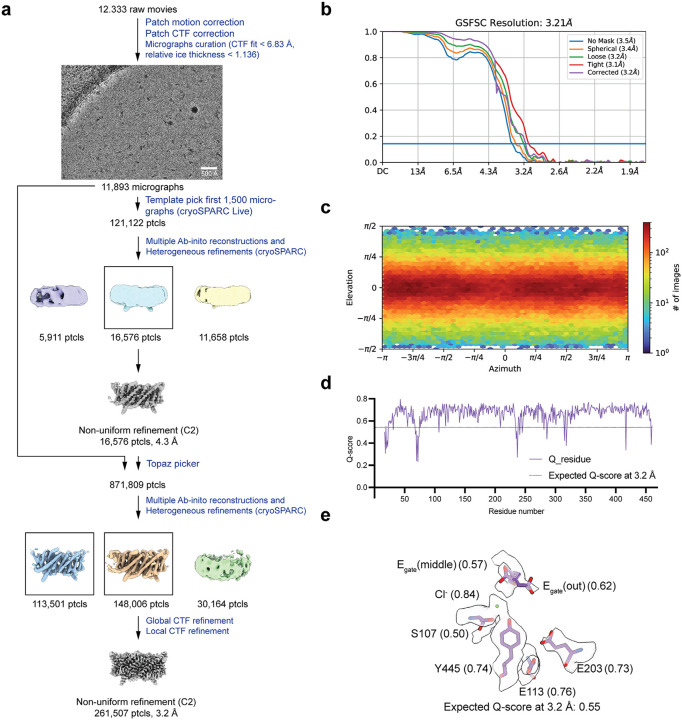
Cryo EM workflow and validation data for CLC-ec1 at pH 4.0 (**a**) Cryo-EM data processing workflow. (**b**) Gold-standard FSC curve. The resolution is estimated based on FSC at 0.143. (**c**) Angular distribution plot (**d**) Per-residue Q-score as a function of residue number. The expected Q-score at the map resolution is indicated by the dotted line. (**e**) The cryo-EM density and molecular model overlay for central Cl^‒^ binding site. The key residues and bound Cl^‒^ and their corresponding Q-score are annotated.

**Extended Data Figure 6:**
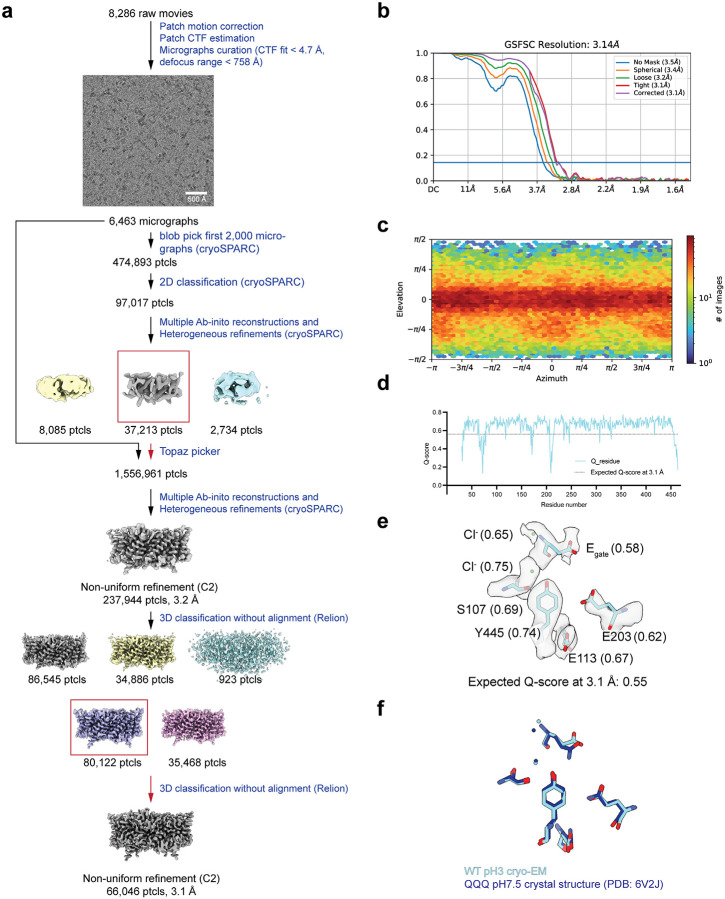
Cryo EM workflow and validation data for CLC-ec1 at pH 3.0. (**a**) Cryo-EM data processing workflow. (**b**) Gold-standard FSC curve. The resolution is estimated based on FSC at 0.143. (**c**) Angular distribution plot (**d**) Per-residue Q-score as a function of residue number. The expected Q-score at the map resolution is indicated by the dotted line. (**e**) The cryo-EM density and molecular model overlay for central Cl^‒^ binding site. Key residues and bound Cl^‒^ and their corresponding Q-score are annotated. (**f**) Overlay of our cryo-EM structure (cyan) with the previous QQQ crystal structure (dark blue) (PDB: 6V2J).

To visualize these pH-dependent changes at high resolution, we determined cryo-EM structures of CLC-ec1 at pH 7.5, 4.0, and 3.0, as summarized in Fig. 3 and Table 1. Notably, the E_gate_ was well-resolved in all three structures, as evidenced in the density maps and by the Q-scores^55^ (Extended Data Figs.4–6). In the pH 7.5 structure (3.18 Å resolution), E_gate_ occupies the S_ext_ anion-binding site in the “middle” orientation (Fig. 3a,d, Extended Data Fig. 4). This structure resembles the original CLC-ec1 crystal structure (1OTS, 2.51 Å resolution^23^), with a C-α r.m.s.d of 1.00 Å. In the pH 4.0 structure (3.21 Å resolution), we observed a mixture of E_gate_ in both the “middle” and “out” orientations, suggesting a transitional phase (Fig. 3c,e, Extended Data Fig. 5). In contrast, at pH 3.0 (3.14 Å resolution), E_gate_ is observed only in the “out” orientation (Fig.3c,f, Extended Data Fig. 6). These results confirm the pH-dependent modulation of E_gate_ conformations.

In the pH 4.0 cryo-EM structure, the primary conformational change compared to pH 7.5 is an outward shift of helices A and G-H-I, which opens access to the H^+^ permeation pathway (Fig. 3g). These changes at pH 4.0 compared to pH 7.5 are consistent with the increased water accessibility observed by HDX at pH 4.0 (Extended Data Fig. 2) and were also seen in the published pH 4.5 cryo-EM structure of CLC-ec1^25^.

In the pH 3.0 cryo-EM structure, there is a subtle conformational change compared to pH 4.0, with an overall C-α r.m.s.d of 0.92 Å. Notably, the pH 3.0 conformation resembles the “QQQ” CLC-ec1 crystal structure (6V2J) (overall C-α r.m.s.d 0.84 Å) more closely than the pH 4.0 structure (overall C-α r.m.s.d 1.27 Å) (Fig. 3h). Additionally, while the pore radius profiles of the pH 3.0 and 4.0 structures are similar to one another (Fig. 3i), at pH 3.0 E_gate_ is shifted towards the pore, and residue F357, which in simulations lowers the energy barrier for Cl^‒^ movement^38^, is observed in the energy-lowering “up” position (Fig. 3j). Together, these subtle conformational changes at pH 3.0 vs 4.0 motivated us to perform molecular dynamics simulations on the pH 3.0 structure.

### Molecular dynamics simulations reveal water wires, Cl^‒^ leaving, and inner-gate opening

**Figure 1:**
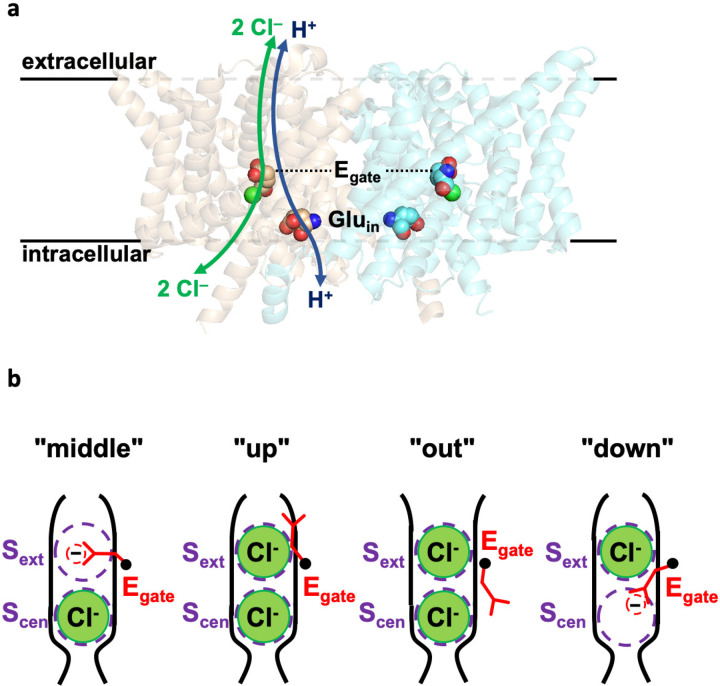
CLC structure overview. (**a**) Side view of CLC-ec1 (pdb 1OTS). The Cl^‒^ and H^+^ permeation pathways are indicated by green and blue arrows, respectively. Bound Cl^‒^ is shown as a green sphere. Key glutamate residues E_gate_ and Glu_in_ are shown space filled. E_gate_ physically gates the Cl^‒^ permeation pathway and serves as a conduit for H^+^; Glu_in_ lines the intracellular portion of the H^+^ permeation pathway. (**b**) Cartoon depictions of the Cl^‒^ pathway, showing the conformations of E_gate_ in high-resolution structures: “middle” (CLC-ec1^19^), “up” (CLC-ec1^23^, CLC-7^92^), “out” (CLC-ec1^24,25^, CLC-1^93^), and “down” (cmCLC^94^, CLC-2^95^).

**Fig. 2:**
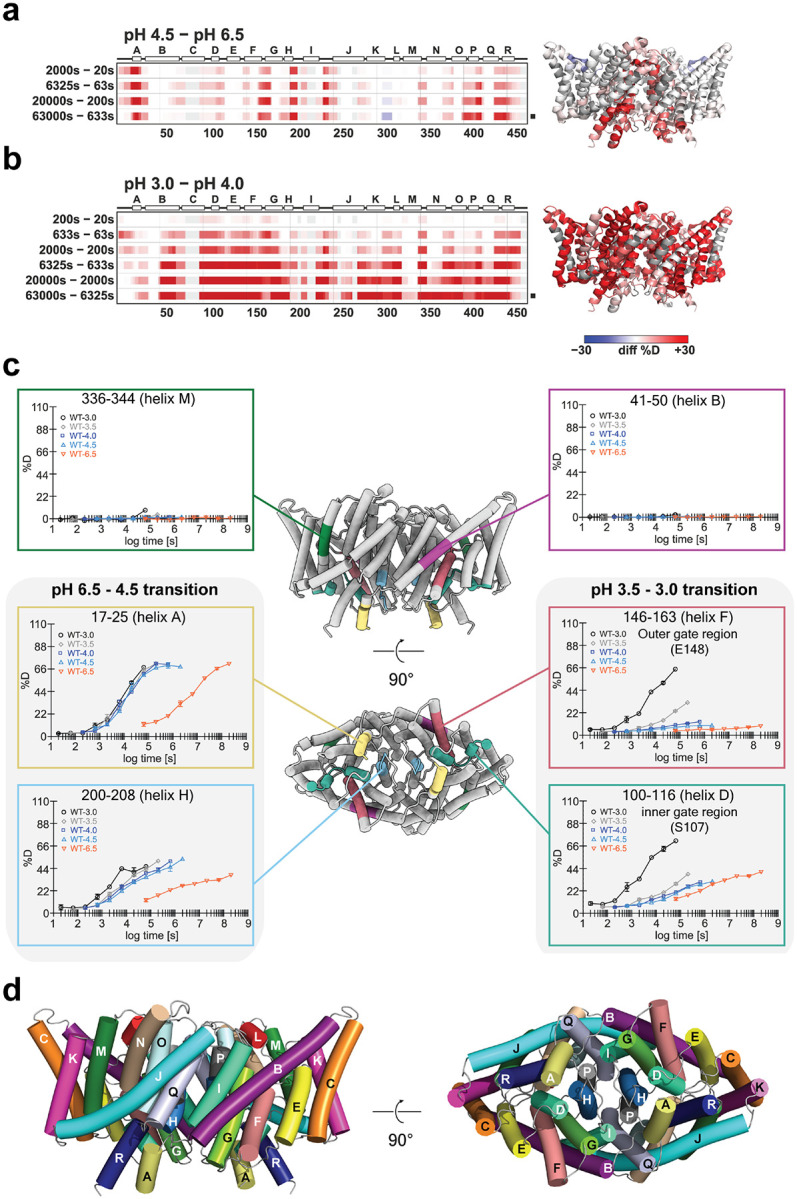
HDX-MS analysis of CLC-ec1 reveals dynamics at low pH. The pH-driven structural transitions as captured by HDX-MS. (**a**) Deuteration levels of CLC-ec1 at pH 4.5 were subtracted from those obtained at pH 6.5 and the results were visualized via a differential heat map. Each colored line represents a time point of the HDX kinetics. Due to differences in the chemical exchange rate at pH 4.5 and 6.5 (100-fold), time points with equal exchange were compared (e.g. 20s at pH 6.5 vs. 2000s at pH 4.5).The blue-white-red color gradient indicates deuteration levels. Differences at the final time point (denoted by a black square dot on right of the heat map) were plotted onto the CLC-ec1 structure at right. (**b**) Differential heat map capturing the time resolved HDX kinetics of the second transition, shown for time points of matching exchange (10-fold difference due to 1 pH unit shift). The last timepoint was used for CLC-ec1 structure coloring. (**c**) Selected deuterium uptake plots. The peptides monitored are indicated at the top of each plot and are mapped by color onto the CLC-ec1 structure at center (views from the perspective of the membrane and from the intracellular side). Exchange times are normalized to pH 3.0 to compensate for the differences in exchange rates. For replicated time points (20s, 633s and 20000s), error bars indicate standard deviations. Data were corrected for back-exchange. Graphs at left and right depict representative peptides that capture major pH transitions. Regions of no or small pH-induced changes are represented by the graphs at the top. The full set of uptake plots are available in Extended Data Figure 3. (**d**) Membrane and intracellular views of CLC-ec1 with helices labeled.

Connected water wires can facilitate proton conduction via a Grotthuss mechanism^56,57^. Early simulations on CLC-ec1 showed water wires transiently connecting E_gate_ to Glu_in_^32–39^, which is a residue near the intracellular side of the transmembrane domain (Fig. 1a) whose neutralization has a major impact on H^+^ transport^24,31^. More recently, water wires connecting E_gate_ directly to the intracellular solution were observed in MD simulations of the QQQ mutant crystal structure^24^ and in simulations of WT CLC-ec1 pH 4.5 cryo-EM structure^25^. These water wires provide a plausible explanation for the transfer of H^+^ to and from E_gate_ when it is in the “out” position. However, they do not account for all H^+^ transport steps needed to model a complete, reversible Cl−/H+ transport cycle, including how E_gate_ becomes protonated when it is in the “down” position within the anion pathway. Simulations of the new pH 3.0 cryo-EM structure could provide further insights into this question or shed light on other aspects of the transport cycle.

We performed extended molecular dynamics simulations on the pH 3.0 cryo-EM structure in order to evaluate protein dynamics and water-wire formation. We observed three classes of water wires. Two classes (wires 2 and 3) resembled water wires seen in previous simulations, connecting the intracellular solution to E_gate_ in the “out” position, whereas the third class (wire 1) was a novel water wire, connecting the intracellular solution to the anion permeation pathway (Fig. 4a).

Water wires 2 and 3 offer a plausible explanation for how a proton moving to the inside is coupled to two chloride ions moving out, as illustrated in Fig. 4b. Following the delivery of a proton from the “out” E_gate_ to the intracellular solution, the resulting negatively charged E_gate_ will be energetically favored to move from the hydrophobic core of the protein to the anion-friendly pore. To achieve this, E_gate_ must displace any Cl− ions occupying the pore. Since CLCs are antiporters with a strict 2:1 Cl−/H^+^ exchange stoichiometry, the transport of a proton to the intracellular solution would require the deprotonated E_gate_ to displace 2 Cl− ions to the outside (Fig. 4b).

**Extended Data Figure 7:**
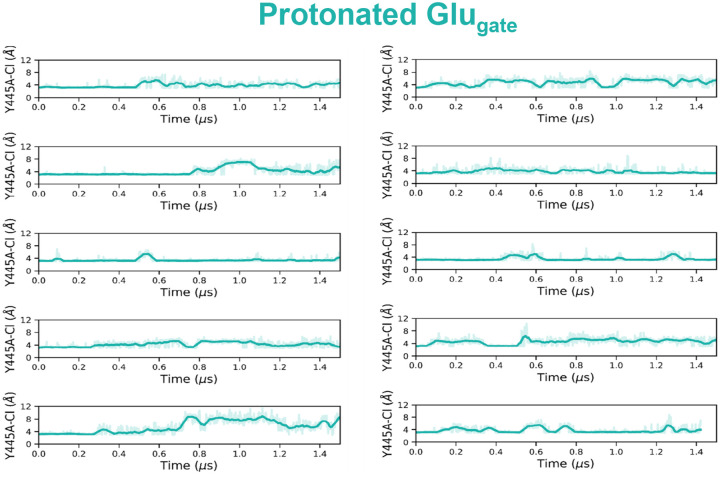
Cl^‒^ remains in the anion pathway during simulations with E_gate_ protonated. The presence of Cl^‒^ within the anion pathway during simulations was evaluated by plotting the distance from the Cα atom of inner-gate residue Y445 to the nearest Cl^‒^ ion.

Capturing the displacement of 2 Cl− ions by E_gate_ in simulations would provide strong support for this proposed water-wire mechanism. We therefore performed simulations on the CLC-ec1 pH 3.0 structure with E_gate_ deprotonated and evaluated the presence of Cl− in the permeation pathway by plotting the distance from pore-lining inner-gate residue Y445 to the nearest Cl− ion. Strikingly, in 8 of 10 independent 1.5-us trajectories, both Cl− ions left the permeation pathway (Fig. 4c). In stark contrast, Cl− remained bound throughout all 10 simulations with E_gate_ protonated (Extended data Fig. 7). Thus, the deprotonation of E_gate_ is a crucial event that triggers expulsion of Cl− from the permeation pathway. Inspection of the trajectories revealed that the Cl− ions always left to the extracellular side, in harmony with the experimentally observed 2:1 Cl−/H^+^ exchange. Surprisingly, movement of E_gate_ into the anion pathway was accompanied by reorientation of inner-gate residues S107 and Y445 (**Movie 1**, Fig. 4d).

### Water wires do not depend on chloride

The binding of Cl^‒^ to the S_cen_ site (Fig. 1b) is recognized as a critical feature in the CLC coupling mechanism and is supported by multiple lines of evidence. First, mutations that weaken Cl^‒^ binding to S_cen_ also reduce coupling efficiency, resulting in fewer H^+^ ions being transported per Cl^‒^ ion^26,40^. Second, polyatomic anions that bind less strongly to S_cen_ than Cl^‒^ also exhibit weaker coupling to H^+ 41^. Finally, Cl^‒^ binding at S_cen_ is critical for water wires that connect Glu_in_ to E_gate_, and mutations that disrupt these water wires reduce coupling efficiency^32,37^. Given these previous findings, we anticipated that the water wires connecting E_gate_ to the intracellular solution would depend on Cl^‒^ binding at S_cen_. To test this hypothesis, we quantified water-wire formation in our two sets of simulations: with E_gate_ protonated (Cl^‒^ always present in the permeation pathway) and with E_gate_ deprotonated (Cl^‒^ absent during >70% of the simulation time). Surprisingly, we found that water wires were similarly abundant in both simulations (Fig. 4e), indicating that water wires do not depend on Cl^‒^ binding.

### Weak Cl^‒^ binding at S_cen_ does not necessarily lead to uncoupling

This finding inspired us to investigate the relationship between Cl^‒^ binding and the coupling mechanism. Prior studies showed that any disruption to anion binding at S_cen_ results in uncoupling^26,40,41^. As a result, it has been reasonably suggested that a key distinction between CLC transporters and channel homologs lies in their Cl^‒^ binding affinity at S_cen_, with CLC channels functioning as uncoupled channels rather than coupled transporters due to their reduced Cl^‒^ binding affinity^27^. But does weak Cl^‒^-binding inevitably result in uncoupling? The mutagenesis studies that established the link between weakened anion binding and uncoupling in CLC transporters involved mutation of residues S107 and Y445. These residues not only directly interact with Cl^‒^ at S_cen_, but they also directly form the inner gate that controls Cl^‒^ passage to and from S_cen_ (4d). Consequently, mutations at these positions physically alter the inner gate, and it is therefore possible that the uncoupling caused by these mutations is more a result of gate disruption than of weakened Cl^‒^ binding.

To test the hypothesis that weak Cl^‒^ binding alone does not cause uncoupling in the CLC transporter, we needed to disrupt Cl^‒^ binding while leaving the inner gate intact. To do so, we focused on K131, as free energy calculations identified it as the most influential side chain in stabilizing Cl^‒^ binding at S_cen_^58^. K131 does not directly interact with Cl^‒^; rather, the positively charged sidechain is buried within the transmembrane domain, with the positively charged sidechain pointing towards S_cen_ and stabilizing Cl^‒^ binding from a distance of 7–9 Å from S_cen_^58^. We first experimentally confirmed weak Cl^‒^ binding by K131A CLC-ec1, as well as the overall structural integrity of this mutant. The cryo-EM structure of K131A CLC-ec1 exhibited minimal differences in overall structure compared with WT CLC-ec1 (overall C-α r.m.s.d 0.52 Å), except for a clear reduction of Cl^‒^ density at S_cen_ (Fig. 5a, Extended Data Fig. 9). Isothermal titration calorimetry (ITC) confirmed weak Cl^‒^ binding by CLC-ec1 K131A (Fig. 5b), with the lack of heat detection indicating Kd > 20 mM^26^.

To evaluate the transport function of CLC-ec1 K131 mutants, we used quantitative ion flux assays on purified transporters reconstituted into phospholipid vesicles^59^ (Fig. 5c). We examined both K131A (for which we have structural and ITC data) and K131M, which was previously studied using planar lipid bilayer recordings^31^. We found that the unitary Cl^‒^ turnover rate (Cl^‒^ ions/s per transporter) was greatly reduced in K131 mutant transporters compared to WT (Fig. 5d,e and Table 2). Their unitary H^+^ turnover rates were also greatly reduced, to levels just measurable above background (Fig. 5d,e and Table 2). Importantly, we structured this flux-assay experiment to ensure that any H^+^ movement into the vesicles was a result of Cl^‒^-dependent H^+^ pumping rather than leakage (Fig. 5c). Therefore, the low H^+^ transport rates measured for K131 mutant transporters represent *bona fide* H^+^ pumping rates. By analyzing the ratio of the Cl^–^ and H^+^ transport rates, we determined the Cl^–^/H^+^ transport stoichiometry (and thus the strength of coupling) for WT and mutant CLC-ec1 transporters. Strikingly, coupling in the K131 mutant transporters was largely intact, with a just a slight increase in Cl−/H^+^ transport stoichiometry from ~2 for WT to ~3 for both K131 mutants (Fig. 5f, Table 2). This modest reduction in Cl^–^/H^+^ coupling efficiency is consistent with findings from K131M studied in planar lipid bilayers, where shifts in reversal potentials suggested slight uncoupling^31^. Together, these results illustrate that weak binding at S_cen_ does not on its own cause uncoupling.

The dramatic slowdown in transport rates by K131A and K131M mutant transporters (Fig. 5d,e) could be due directly to their weak Cl^–^ affinity, with Cl^–^ binding being rate limiting for transport. Alternatively, it could be due to an indirect effect, with decreased Cl^–^ binding at S_cen_ causing the K131 transporters to stall at one or more of the H^+^-transport steps in the cycle. To distinguish these possibilities, we evaluated the effect of K131 mutations in the background of the E_gate_ mutation, E148A. CLC-ec1 E148A, lacking a titratable residue at the critical E_gate_ position, is completely unable to transport H^+^ and operates strictly as a passive Cl^–^ transporter^1^. If K131 mutations slow transport because they weaken Cl^–^ binding, then they should affect Cl^–^ transport rates in both WT and E148A backgrounds. On the other hand, if K131A mutations slow transport because they cause the transporter to stall at one of the H^+^-transport steps, then they should have no effect in the E148A background, where there are no H^+^-transport steps. We therefore tested CLC-ec1 K131A/E148A and CLC-ec1 K131M/E148A mutant transporters using the quantitative Cl^–^ flux assay (Fig. 5c) (measuring only Cl^–^ since E148 transporters do not transport H^+^). K131 mutations had no discernable effect on E148A Cl^–^ transport rates (Fig. 5g, Table 2). These results align with the “stalled transport cycle” model for K131 mutants: when these low Cl^–^-binding mutant transporters are untethered from obligate Cl^‒^/H^+^ coupled transport, Cl^–^ movement becomes faster, unimpeded by the need to wait for a stalled proton-transport step.

## DISCUSSION

A comprehensive model to explain the CLC transporters’ 2:1 Cl^‒^/H^+^ exchange mechanism has been elusive. This study was conducted with the hypothesis that investigating CLC conformational dynamics across a broader expanded pH range – encompassing the physiological pH associated with maximal activity – would yield new insights into the exchange mechanism.

### Conformational Dynamics at pH 3.0

**Figure 3:**
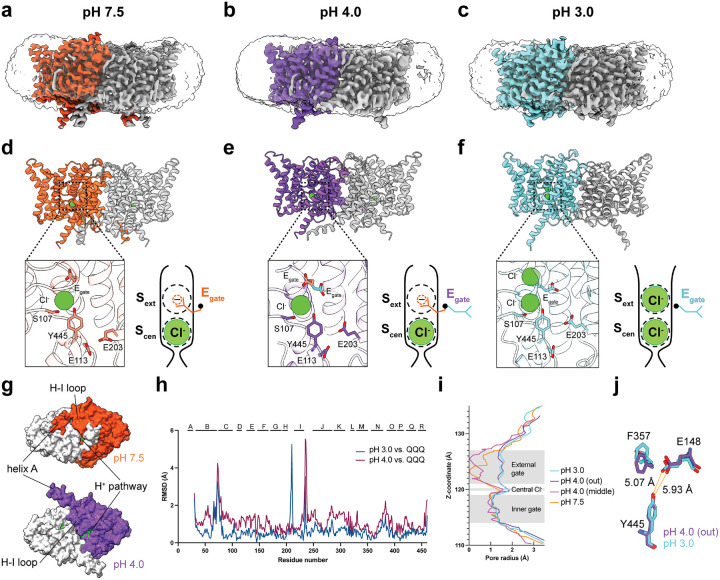
Cryo-EM structures of CLC-ec1. (**a, b, c**) Cryo-EM density maps for CLC-ec1, with each panel showing one of the two subunits in color: pH 7.5, orange; pH 4.0, purple; pH 3.0, cyan. (**d, e, f**) Molecular models of CLC-ec1 for structure determination at pH 7.5, 4.0, and 3.0, with colors as in panels a-c. The zoomed-in views of the models showing key residues and resolved Cl^−^ ions. Cartoons depictions to the right of the zoomed-in views are as in Fig. 1, highlighting the E_gate_ conformations observed at pH 7.5 (“middle” conformation), pH 4 (“middle” and “out” conformations), and pH 3 (“out” conformation). (**g**) View from the intracellular side, highlighting major conformational changes between pH 7.5 and 4.0. Residues lining the H^+^ pathway are colored in green (E113, E148, A182, L186, A189, F190, F199, E202, E203, M204, I402, T407 and Y445), to highlight the opening of this pathway in the pH 4.0 conformation. (**h**) Comparison of pH 4.0 and pH 3.0 cryo-EM structures to the “QQQ” crystal structure. The structural alignment was done in ChimeraX using the matchmaker command aligning the dimer. (**i**) Pore radius profiles calculated using HOLE. (**j**) Zoomed-in view comparing E148 and F357 between pH 4 (out) and pH 3. The inner gate residue Y445 is shown for reference.

Previous spectroscopic and cryo-EM studies revealed that the CLC-ec1 transporter undergoes conformational change at pH 4.5 in comparison to pH 7.5 and that this conformational change is essential for activity^25,30^. Our HDX-MS results align with those previous results and reveal additional conformational dynamics that occur at lower pH values, down to pH 3.0. Our cryo-EM structure at pH 4.0 reveals a conformational change that opens access to the H^+^ permeation pathway (Fig. 3g), similar to that reported in cryo-EM studies of CLC-ec1 at pH 4.5^25^. At pH 3.0, we observe further conformational changes, particularly involving residues near the ion permeation pathways (Fig. 3h,j).

In a recent cryo-EM study on CLC-ec1 by Fortea et al., a “Twist” conformation featuring a notable 28.4-degree rotation between subunits was resolved at pH 4.5^25^. Interestingly, we did not observe this conformation in our pH 4.0 dataset. Our initial hypothesis was that the difference might stem from the membrane mimetics used: we employed Salipro lipid nanodiscs, whereas Fortea et al. used the detergent decyl maltoside (DM). To test this hypothesis, we determined a cryo-EM structure under our original conditions (pH 4.0, 150 mM NaCl) but using DM instead of Salirpo nanodiscs. However, we still did not detect the “Twist” conformation; thus, the difference in membrane environment does not account for our lack of detection of the Twist conformation. Other differences between our experimental preparation and that of Fortea et al. include pH (4.0 vs 4.5), NaCl concentration (150 mM vs 100 mM), and buffer (citrate vs acetate). Given that the Twist conformation is not essential for function (preventing its formation with cross-links has no effect on activity^25,28^), it is perhaps not surprising that it is not detected under all conditions that support activity.

### Conformational dynamics revealed by molecular dynamics simulations

**Figure 4:**
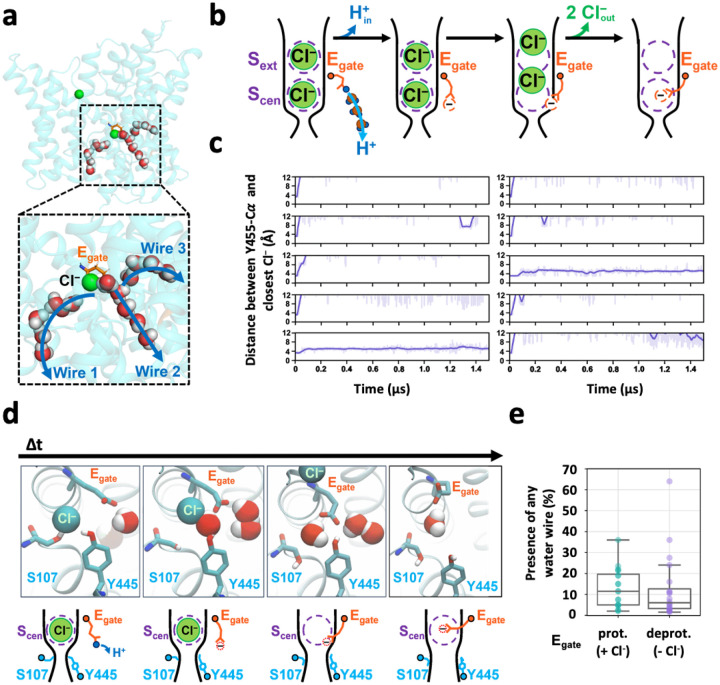
Molecular Dynamics simulations reveal water wires, Cl^−^ leaving, and inner-gate opening. (**a**) Simulation snapshot depicting water wires that connect to the intracellular solution. (**b**) Cartoon model of proton transfer from E_gate_ to the intracellular solution along water wire 2. The resulting negatively charged E_gate_ is predicted to return to the anion pathway, expelling two Cl^‒^ ions. (**c**) Cl^‒^ leaves during 8 out of 10 1.5-microsecond simulations with E_gate_ deprotonated. Each plot shows the distance from inner-gate residue Y445 to the nearest Cl^‒^ ion; >12 Å indicates that Cl^‒^ no longer occupies anion binding sites with the pathway. Unsmoothed traces (light purple lines) and traces smoothed with a moving average (dark purple lines) are shown for all simulations. The time traces were smoothed using a moving average with a window size of 20 ns. (**d**) Simulation snapshots showing a representative Cl^‒^-leaving event. Concomitant with Cl^‒^ leaving to the extracellular side (i.e., away from the viewer), inner-gate residues S107 and Y445 move away from one another, opening the inner gate. Cartoon depictions are shown beneath each snapshot. (**e**) Water wires occurred with similar abundance in E_gate_ protonated and deprotonated simulations, indicating that Cl^‒^ binding does not substantially influence water-wire formation. Each dot corresponds to one simulation and shows the fraction of frames in which any of the three classes of water wires is formed. The middle line of each box in the plot is the median across simulations, with the box extending from the 1^st^ to the 3^rd^ quartile and defining the interquartile range. Whiskers extend to last data points that are within 150% of the interquartile range. Note that Cl^‒^ is always bound in simulations with E_gate_ protonated, whereas no Cl^‒^ is bound in over 70% of simulation frames with E_gate_ deprotonated. A simulation snapshot showing water wires in the absence of Cl^‒^ is shown in Extended Data Fig. 8.

Our molecular dynamics simulations on the pH 3.0 cryo-EM structure provided unprecedented visualization of key steps in the Cl^‒^/H^+^ exchange mechanism. With E_gate_ deprotonated, we dynamically observed the conformational change of E_gate_ from the “out” to the “down” position. Remarkably, this conformational change occurred in concert with the displacement of two Cl^‒^ ions into the extracellular solution, directly illustrating CLC-ec1’s 2:1 exchange stoichiometry. Additionally, the accompanying reorientation of inner-gate residues S107 and Y445 (**Movie 1**, Fig. 4d) was an unexpected observation that provides further clarity on the exchange mechanism. This reorientation unveils a long-awaited snapshot of the “inward-open” conformational state of CLC transporters.

Our simulations revealed water wires that connect the intracellular solution to the CLC-ec1 protein core. These wires occur both through the canonical H^+^ pathway, as have been reported previously^24,25,32–39^ and through the Cl^‒^ pathway (Fig. 4a). Surprisingly, these water wires occur regardless of whether Cl^‒^ is bound (Fig. 4e), contradicting prior simulations that indicated water wires depend on Cl^‒ 32,37^. However, the prior simulations were conducted on a pH-7 structure now thought to represent an inactive or poorly active conformation^25^. Additionally, the water wires observed in prior simulations connected E_gate_ only to Glu_in_, which is beneficial but non-essential for H^+^ transport^24^, and not to the intracellular solution. Nonetheless, the simulation result is surprising because the water wires that form in the absence of Cl^‒^ appear to create a pathway for uncoupled transport. In this scenario, H^+^ transfer from the intracellular solution to E_gate_ could be followed by movement of protonated E_gate_ to the “out” position, allowing a proton to be transferred to the extracellular solution without concomitant Cl^‒^ movement. However, the risk of uncoupled proton occurring in the Cl^‒^-free transporter may be alleviated if the protonation energy for E_gate_ is excessively high in the absence of Cl^‒^. Indeed, free energy calculations conducted on CLC-ec1 in its low-activity state have predicted this phenomenon, with experimental ion-binding studies supporting the computational prediction^27^. Therefore, the observed abundance of water wires, regardless of the presence of Cl^‒^, does not inherently pose a risk to coupling.

### The mechanism of reversible Cl^‒^/H^+^ exchange

Our findings, combined with previous research, provide an unprecedented opportunity to propose a complete and efficient CLC 2:1 Cl^‒^/H^+^ exchange mechanism, free from steps that are energetically unviable (Fig. 6). The transport mechanism involves conformational states identified in structural studies— “up,” “out,” “down,” and “middle”—alongside transient states observed in simulations. The cycle, which can proceed in either direction, facilitates the transmembrane exchange of two Cl^‒^ ions for one H^+^, with each step characterized by energetically plausible mechanisms. Beginning at the top left with E_gate_ in the “up” conformation (state **A**) and moving through the cycle clockwise, protonation from the extracellular solution enables the transition to the “out” conformation, with E_gate_ in the hydrophobic core of the protein (state **B**). From there, water wires facilitate transfer of a proton to the intracellular solution. (For simplicity of illustration, we show only one water wire, though multiple water wires may form.) The resulting negatively charged E_gate_ moves from the hydrophobic core of the protein into the anion-friendly pore (transient state **C**), expelling Cl^‒^ to the outside, either two ions at once, as observed in our simulations, or one at a time, as modeled here (states **D** and **E**). From the single-site occupancy states (**D** and **E**), which have been observed crystallographically (“down” and “middle”), the transient opening of the inner gate, as observed in our simulations, provides a pathway for Cl^‒^ to enter from the intracellular solution. From state **E**, Cl^‒^ entry expels E_gate_ to the “up” conformation, allowing it to be protonated from the extracellular side, thus initiating another transport cycle. All steps in this mechanism are inherently reversible.

Notably, the newly identified water wire (Wire 1 in Figure 4, illustrated here in states **C** and **D**), which connects the intracellular solution to E_gate_ positioned in the “down” conformation, offers a physically plausible pathway for the transport cycle to proceed in the counterclockwise direction, with Cl^‒^ ions moving inward and H^+^ ions moving outward. Starting at the top left in state **A** and moving counterclockwise, the negatively charged E_gate_ moves into the Cl^‒^ pathway, displacing a Cl^‒^ ion to the intracellular side and generating state **E**. Subsequent entry of a Cl^‒^ ion from the extracellular side knocks E_gate_ to the “down” position and displaces a second Cl^‒^ ion, generating state **D.** Water wires can connect to E_gate_ in this conformation, but protonation of E_gate_ is not energetically favorable at this stage, with only 1 Cl^‒^ ion in the pathway. However, when a second Cl^‒^ enters (state **C**), protonation of E_gate_ becomes energetically favorable. Following protonation, the neutralized E_gate_ is favored towards the “out” conformation (state **B**). A subsequent shift to the “up” conformation (state **A**) enables release of a proton to the extracellular solution, thereby completing the transport cycle in that direction.

**Figure 5:**
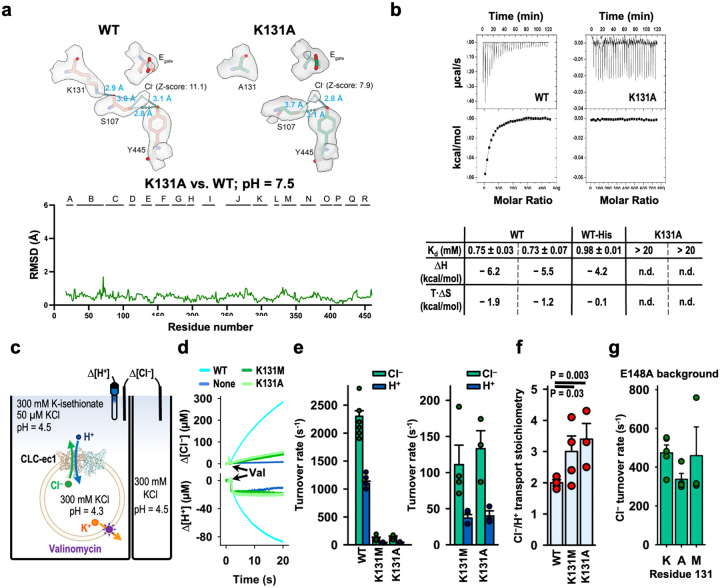
K131 mutation reduces Cl^‒^ binding at S_cen_ but maintains Cl^‒^/H^+^ coupling. (**a**) Comparison of K131A and WT CLC-ec1 cryo-EM structures. Top panels: Region around the S_cen_ site. The Cl^–^ signal at S_cen_ is substantially less distinct in K131 compared to WT (7.9 vs 11.1 sigma z-score^96^). Bottom panel: The overall structures are highly similar (RMSD = 0.52 Å). Structural alignment was done in ChimeraX using the matchmaker command aligning the dimer. (**b**) Cl^–^ binding measured by ITC for WT and K131A CLC-ec1. The top panels show representative primary data from ITC experiments. The summary table below shows results for experiments performed on 3 (WT) or 2 (K131A) separate protein preparations; for K131, no heat of binding was detected (n.h.d.) in either preparation. (**c**) Cartoon depiction of the functional assay to quantify H^+^/Cl^–^ transport rates and coupling stoichiometry. Extravesicular [Cl^–^] and [H^+^] were simultaneously measured using Ag/AgCl and pH electrodes, respectively. Because the Cl^–^/H^+^ exchange cycle nets three charges across the membrane per cycle, buildup of an electrical gradient means that measurable Cl^–^ or H^+^ transport does not occur until the addition of valinomycin, which dissipates the electrical gradient by shuttling K^+^ ions. The experimental setup involves a 2-fold gradient for H^+^, such that any leak will involve movement of H^+^ out of the vesicles, and any H^+^ movement into the vesicles must occur via CLC-ec1 actively transporting H^+^ via coupling to the downhill movement of Cl^–^. (**d**) Representative Cl^‒^ and H^+^ traces (upper and lower panels, respectively) for flux assays performed on vesicles reconstituted with WT, K131A, K131M, or no CLC-ec1 protein. “Val” indicates the time of addition of valinomycin. The signals indicate net Cl^–^ release from vesicles and net H^+^ uptake into the vesicles. (**e**) Summary of transport rates for WT, K131A, and K131M CLC-ec1. The graph on the right shows the data using an expanded scale for the y-axis. (**f**) Summary of Cl^–^/H^+^ coupling stoichiometry of WT and K131 mutant transporters. P values were determined using Student’s t-test. (**g**) Summary of flux assays on uncoupled transporters (E148A background) with K (WT), A, or M at the 131 position, showing that K131 mutations do not affect turnover rates of uncoupled transporters.

**Figure 6:**
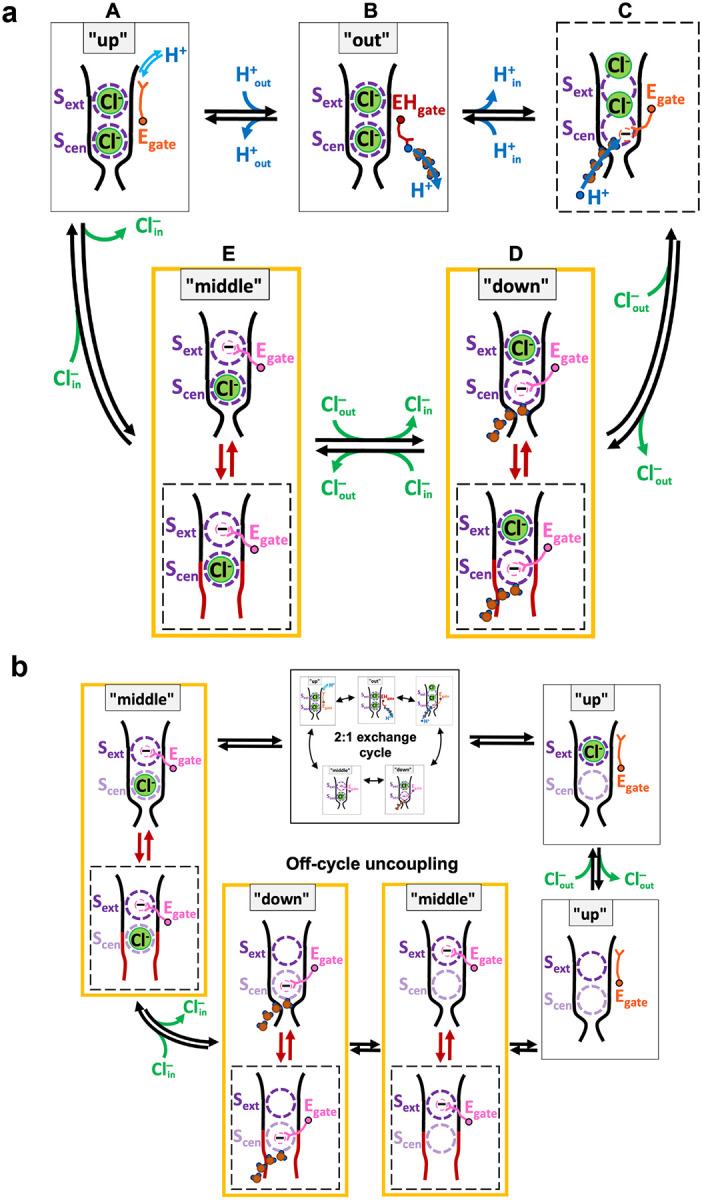
Model for reversible Cl^‒^/H^+^ exchange by CLC transporters. (**a**) The 2:1 stoichiometric transport mechanism involves conformational states identified in structural studies (labeled “up”, “out”, “down”, and “middle”, shown in solid boxes) along with transient states observed in simulations (illustrated within dashed boxes). For clarity, water wires are shown only at key H^+^-transport steps. The cycle, which can proceed in either direction, facilitates the transmembrane exchange of two Cl^‒^ ions for one proton (H^+^). A detailed description of the model can be found in the main text. (**b**) In K131 mutant transporters that have weakened binding at S_ext_ and S_cen_, off-cycle transitions allow Cl^‒^ transport independent of H^+^ transport. The mild uncoupling observed experimentally (3:1 Cl^‒^/H^+^) can be accounted for by one off-cycle event occurring during each regular transport cycle.

K131 is universally conserved across the CLC family, including both transporter and channel homologs. This conservation, combined with K131’s location - entirely buried within the transmembrane domain, which is rare for lysine residues^60^ – suggests that it plays a crucial functional role. Indeed, mutations at this position in CLC channels shift voltage dependence and lead to myotonia^61–64^. In CLC-ec1, we found that K131 mutations weaken Cl^‒^ binding affinity and reduce Cl^‒^/H+ transport rates (Fig. 5, Table 2). The exchange mechanism illustrated in Fig. 6a provides a framework for understanding how this weakened Cl^‒^ binding results in reduced Cl^‒^/H+ transport rates. In this context, the impact of reduced Cl^‒^ binding at S_ext_ and S_cen_ must be considered relative to E_gate_ binding at these sites. Although E_gate_ binding affinity may also be weakened in the mutants, its physical tethering gives it a competitive advantage over free Cl^‒^ ions. Supporting this idea, the K131A structure shows that the cryo-EM density for E_gate_ is less affected compared to that of Cl^‒^. Consequently, Cl^‒^ will struggle to compete with E_gate_ for the S_ext_ and S_cen_ sites, thereby slowing the D⇄E, D→C, and E→A transitions and stalling the transport cycle.

In addition to reducing Cl^‒^ binding and transport rates by more than tenfold, K131 mutations cause mild uncoupling, increasing the Cl^‒^/H^+^ stoichiometry from 2 to ~3. A potential mechanism for this experimentally observed uncoupling may involve off-cycle transitions through states with unoccupied anion-binding sites, as illustrated in Fig. 6b. During these off-cycle transitions, weakened Cl^‒^ binding allows Cl^‒^ to exit the permeation pathway independently, without being displaced by incoming Cl^‒^ from the opposite side. As a result, these off-cycle transitions facilitate the transport of a Cl^‒^ ion independently of H^+^ transport. Within this context, Cl^‒^ movement to and from the interior is likely facilitated by a slight weakening of the inner gate, due to the loss of hydrogen bonds between K131 and the conserved inner-gate loop residues A104 and G106. Nevertheless, since the uncoupling is mild, these off-cycle events do not predominate but rather occur approximately once per transport cycle.

The proposed transport mechanism (Fig. 6a) is relevant to CLC transporters across all kingdoms, which share fundamental features including 2:1 anion/proton coupling and E_gate_ as an essential coupling element^4^. The mechanism elucidates how transport occurs in the direction of Cl^‒^ ions moving inward across the membrane and H^+^ ions moving outward, which is the direction of physiological relevance for CLC-ec1 in facilitating extreme acid tolerance^15^ and for mammalian CLCs in acidifying intracellular compartments^22,65^. While this mechanism addresses the fundamental Cl^‒^/H^+^ exchange function, questions remain concerning how this core function is regulated in the various CLC transporters. For example, mammalian CLCs contain large cytoplasmic domains that mediate regulation by signaling lipids^65–67^ and by nucleotides^68–71^, but these regulatory mechanisms are only partially understood. Many prokaryotic homologs also feature such domains, although their functional roles have yet to be investigated. Gaining insight into how these regulatory mechanisms interact with the core Cl^‒^/H^+^ exchange process will be crucial for fully understanding the diverse functions of CLC transporters in cellular physiology.

## METHODS

### Mutagenesis

Mutations were introduced into the WT CLC-ec1 gene in pASK^17^ using New England BioLabs Q5 Site Directed Mutagenesis kit (New England Biolabs, Ipswich, MA, USA). Each mutation was subsequently verified with whole plasmid sequencing and analyzed using SnapGene^®^ software 2023 (from Dotmatics; available at snapgene.com).

### Protein Production and Purification

CLC ec-1 with an N-terminal polyhistidine tag, in the pASK90 expression vector^72^, was transformed into BL21 *E. coli* cells and plated onto LB plates containing 100 μg/mL ampicillin. The following morning, the colonies from 1–2 plates were scraped into 10 mL LB, added to 1 L of Terrific Broth^73^ in a 2.8-L baffled flask, and cultured at 37°C with vigorous shaking (~220 RPM). When the culture reached 1.0 OD_600_, it was induced with 0.2 mg/L anhydrotetracycline (added from a 0.2 mg/mL solution in dimethylformamide). The cultures were grown for an additional 3 hours then pelleted at 2246*xg* for 20 minutes and flash-frozen in liquid nitrogen for future purification.

For purification, CLC-ec1 pellets were extracted with 50 mM n-Decyl-α-D-Maltopyranoside (DM) (Anatrace, sol grade) for two hours then centrifuged at 25,759*xg* for 45 minutes. The protein was then isolated using a cobalt affinity chromatography step (3 mL of 50% cobalt metal affinity resin slurry, Takara Bio USA, Inc. San Jose, CAl for each 1L prep), eluting with cobalt wash buffer (200 mM NaCl, 20 mM DM (sol grade), 400mM Imidazole, 40 mM TrisCl, pH 7). The eluate was incubated with Endoproteinase Lys-C (Santa Cruz Biotechnology, Inc.), 0.17 U per L culture. The final purification by size exclusion chromatography (SEC) (Enrich SEC 650 10 × 300 column, Bio Rad Laboratories Pleasanton, CA) was carried out in SEC Buffer (10 mM NaHepes, 150 mM NaCl, 5 mM DM (anagrade), pH 7.5). Purified protein was concentrated using Amicon^Ⓡ^ Ultra-15 centrifugal filters with a 50-kD cut-off.

For single-particle cryo-EM experiments at pH 7.5 and 4.0, CLC-ec1 purified by SEC as above, was reconstituted into Saposin-lipoprotein nanoparticle (Salipro (https://doi.org/10.1038/nmeth.3801)) at either pH 7.5 and pH 4. We followed the reconstitution protocol described in Chien et al.^74^ with some modifications. For the pH 7.5 Salipro sample preparation, 0.5 mg of CLC-ec1 in DM at pH 7.5 was mixed with 1.35 mM of POPE, 450 μM of POPG, 0.2 % (w/v) of DDM, 120 μM of Saposin-A, and Gel-filtration buffer (GFB) (10 mM Hepes, 150 mM NaCl, pH 7.5) to a total volume of 2.5 mL. The mixture was incubated at 4 °C for 45 min. Then 50 % (w/v) of Amberlite XAD-2 (Millipore Sigma) was added to the mixture followed by 15 min incubation at 4 °C. The Amberlite XAD-2 beads were removed by centrifugation. The mixture was concentrated to 200 μL using a 10 kDa cutoff Amicon (Millipore Sigma). A final SEC step was carried out using Superdex 200 Increase (10/300) (Cytiva) equilibrated in GFB. The fractions corresponding to CLC-ec1 in Salipro were collected and concentrated using a 3-kDa cutoff Amicon concentrator (Millipore Sigma) to a final concentration of 2.2 mg/mL. For the pH 4.0 Salipro sample, 1.35 mM of POPE, 450 μM of POPG, 0.2 % (w/v) of DDM, 120 μM of Saposin-A and the pH 4 buffer (1.8 mM Citric Acid, 8.2 mM Sodium Citrate, 150 mM NaCl, pH 4) were mixed first and incubated at 37 °C for 10 min. Then 1.5 mg of CLC-ec1 in DM at pH 7.5 was added to the mixture making a total volume of 5 mL. The mixture was incubated at 37 °C for 5 min. The rest of the steps including Amberlite XAD-2 beads and gel-filtration are the same as for the Salipro pH 7.5 sample.

For cryo-EM of WT CLC-ec1 at pH 3, protein was generated using the method as described above but with modification to generate a final sample in the detergent lauryl maltose neopentyl glycol (LMNG). During the cobalt-affinity chromatography step, DM gradually replaced with LMNG (Anatrace), to a final concentration of 20 μM LMNG. The exchange was as follows 50:50, 25:75, 10:90, and finally 5:95 percent by volume DM to LMNG. The final SEC step was performed in pH 3 buffer, 8.2mM Citric Acid, 1.8mM sodium Citrate, 150mM NaCl and 20 μM LMNG. For cryo-EM of K131A, the LMNG protocol was used as described above, except the pH in the final SEC buffer was 7.5 (10 mM NaHepes, 150 mM NaCl, 20 μM LMNG, pH 7.5).

### Hydrogen/deuterium exchange mass spectrometry

For HDX experiments, CLC-ec1 purification was done using the standard protocol with a final SEC step in 7.5 mM HEPES, 100 mM NaCl, 1.8 mM DM (anagrade), pH 7.5. This stock was then buffer exchanged to H_2_O buffer of different pHs, incubated afterwards at least 1hr and then used to run HDX.

For HDX, McIlavaine buffers of the desired pH were prepared. Solution A (0.2 M Na_2_HPO_4_) and solution B (0.1 M citric acid) were mixed in the recommended proportions for each pH (pH 3.0 – A 206 μl, B 795 μl; pH 3.5 – A 304 μl, B 697 μl; pH 4.0 – A 386 μl, B 615 μl; pH 4.5 – A 454 μl, B 546 μl; pH 6.5 – A 710 μl, B 290 μl). Buffers were diluted 5-fold into water, and then 100 mM NaCl and 1.8 mM DM were added. Buffers were prepared in both H_2_O and D_2_O. The protein was transferred to McIlvaine buffers of pH 6.5, 4.5, 4.0, 3.5, and 3.0 using Zeba Spin 7k MWCO desalting columns (Thermo Scientific).

The pH dependent changes in CLC-ec1 were followed on a detergent solubilized protein at pH 6.5, 4.5, 4.0, 3.5, 3.0. CLC-ec1 in McIlvaine buffer was diluted 5-fold into a D_2_O-based buffer of identical composition and pD. Protein concentration during exchange was 3.2μM and temperature 21 °C. Aliquots were taken at 20s, 63s, 200s, 633s, 2000s, 6325s, 20000s, 63000s, where 20s, 633s, 20000s were triplicated. The exchange was stopped by adding phosphoric acid (mixing ratio 1:1) and freezing in liquid nitrogen. Different phosphoric acid concentrations matching the exchange buffer’s pH and containing 1.8 mM DM were used (pH 6.5 – 60mM, pH 4.5 – 40mM, pH 4.0 – 35mM, pH 3.5 – 30mM, 3.0 – 25mM). Fully deuterated control was prepared to correct for deuterium loss during the analysis.

Each sample was quickly thawed and injected onto a protease column (bed volume 70μl) containing immobilized pepsin and nepenthesin-2^75^. Digestion was done at 19 °C and was driven by a flow of 0.4% formic acid in water delivered by an Agilent 1260 Infinity II Quaternary pump (Agilent Technologies, Waldbronn, Germany) at 200 μL.min-1. Peptides were trapped on a guard column (SecurityGuard^™^ ULTRA Cartridge UHPLC Fully Porous Polar C18, 2.1mm ID, Phenomenex, Torrance, CA) and desalted. Next, separation on an analytical column (Luna Omega Polar C18, 1.6 μm, 100 Å, 1.0×100 mm, Phenomenex, Torrance, CA) was done by an acetonitrile gradient (10%–45%; solvent A: 0.1% FA in water, solvent B: 0.1%FA, 2% water in ACN). Solvents were delivered by the Agilent 1290 Infinity II LC system pumping at 50 μL.min-1. To minimize carry-over and remove the detergent, the protease column was washed by two consecutive injections of 4M urea, 500mM glycine HCl pH 2.3 and 5% acetonitrile, 5% isopropanol, 20% acetic acid in water^76^. The analytical column, together with the guard column, was washed with solvent B for 4 min and then with 70% methanol, 20% isopropanol, and 1% formic acid in water for 4 min. The LC setup was cooled to 2°C to minimize the deuterium loss. The analytical column outlet was directly connected to an ESI source of a timsTOF Pro (Bruker Daltonics, Bremen, Germany) operating in MS mode with 1Hz data acquisition. The data processing started with peak picking in Data Analysis 5.3 and exported to simple text files, which were then further handled in DeutEx.^77^. Data visualization was done using MSTools^78^ and PyMol. Identification of peptides arising from pepsin/nepenthesin-2 digestion was done using the same LCMS system, but the mass spectrometer operated in data-dependent MSMS mode with PASEF enabled. The data were searched using MASCOT (v. 2.7, Matrix Science, London, UK) against a database containing sequences of CLC-ec1, acid proteases, and cRAP.fasta (https://www.thegpm.org/crap/). The search parameters were: precursor tolerance 10 ppm, fragment ion tolerance 0.05 Da, decoy search enabled with FDR <1%, IonScore > 20, and peptide length >5. The mass spectrometry data have been deposited to the ProteomeXchange Consortium via the PRIDE partner repository with the dataset identifier PXD058693.

For the stability control experiment, purified CLC-ec1 in 20 mM HEPES (pH 7.5), 100 mM NaCl, and 1.8 mM DM was transferred to McIlvaine buffer (pH 3.0) containing 100 mM NaCl and 1.8 mM DM using Zeba Spin 7K MWCO desalting columns (Thermo Scientific). After buffer exchange, the protein was incubated for 0, 1, 4, and 8 hours before separation on a size-exclusion chromatography (SEC) column (Enrich^™^ SEC 650, 10 × 300 mm, 24 mL; Bio-Rad). ClC-ec1 maintained at pH 7.5 served as a control. SEC separation was performed using a 1 mL sample containing 1 mg of protein at a flow rate of 1 mL/min. The elution buffer consisted of 20 mM HEPES (pH 7.5), 100 mM NaCl, and 1.8 mM DM. All samples were analyzed under identical conditions.

### Cryo-EM sample preparation, data collection, and image processing

Purified samples with a concentration of 2–4 mg/mL were used for cryo-EM grid preparation. 1 mM fluorinated FOS-choline-8 was added prior to freezing grids to improve ice quality and avoid preferred orientation due to air-water interface interactions. 3 μL of the sample was applied to glow discharged Quantifoil R1.2/1.3 Cu200 grids, then blotted with a Whatman filter paper for 3 s before plunge-frozen in liquid ethane using a Vitrobot Mark IV (Thermo Fisher Scientific) at 4 °C and 100% humidity.

All cryo-EM data were collected with Thermo Fisher Scientific Titan-Krios cryo-electron microscopes operating at 300 keV. The WT pH 7.5 dataset was collected with a Falcon 4 camera without an energy filter. The WT pH 4 dataset and K131A dataset were collected with a Gatan K3 camera and Bio-quantum energy filter set to 20 eV. The WT pH 3 dataset was collected with a Falcon 4 and Selectris energy filter set to 10 eV. Data collection parameters are reported in Table 1. Automated data collection was done using EPU software (Thermo Fisher Scientific).

The complete data processing workflows with specific details for each sample/dataset are reported in Extended Data Figs. 4–6. The overall data processing strategy is similar for all four datasets (WT at pH 7.5, 4, and 3, and K131 at pH 7.5). The raw movies were pre-processed (motion correction and CTF correction) in CryoSPARC live. Micrographs were curated with defined criteria including CTF fit and relative ice thickness. A blob picker or a template picker was used to pick a small subset of the dataset. The picked particles were pruned by 2D classifications, ab-initio reconstructions, and heterogeneous refinements in cryoSPARC. The good particles that could be reconstructed to a map with recognizable protein features were used to train a Topaz particle picker. This Topaz picker was used to pick the full dataset. Several rounds of ab-initio reconstructions and heterogeneous refinements with 3 classes were used to clean the particle stacks. For WT pH 7.5 and pH 3 datasets, 3D classification without alignment in Relion with a mask that covers only the proteins was also used for particle cleanup. The final non-uniform refinement with C2 symmetry imposed was done in cryoSPARC.

### Model fitting and refinement

A CLC-ec1 crystal structure (PDB: 1OTS) was used as an initial atomic model for WT CLC-ec at pH 7.5 and pH 4 and for K131A. The QQQ crystal structure (PDB: 6V2J) was used for pH 3 model building. The initial models were first modified based on our construct (i.e. K131A or WT sequences). These models were rigid-body docked into the cryo-EM map and refined using ISOLDE (10.1107/S2059798318002425) and PHENIX (10.1107/S2059798318006551). All atomic models were validated in PHENIX validation job and Q-score^55^. All structure figures were prepared with UCSF ChimeraX^79^.

### System setup for molecular dynamics simulations

We performed simulations of CLC-ec1 under two conditions: (1) simulations with E148 (Glu_ex_), E203 (Glu_in_), and E113 protonated; (2) simulations with E148 (Glu_ex_) deprotonated, and E203 (Glu_in_) and E113 protonated. We initiated all simulations from a CLC-ec1 structure based on the cryo-EM data reported in this manuscript (specifically, from a model very similar to that presented here but based on an earlier refinement). The 3 Cl^–^ ions bound at S_cen_, S_ext_, and S_int_ anion-binding sites in each of the two subunits were preserved. For each simulation condition, we performed ten independent simulations, each 1.5 μs in length. For each simulation, initial atom velocities were assigned randomly and independently.

For all simulation conditions, the protein structure was aligned to the Orientations of Proteins in Membranes^80^ entry for 6V2J (CLC-ec1 triple mutant (E113Q, E148Q, E203Q)^24^ using PyMOL, and crystal waters from 6V2J were incorporated. Prime (Schrödinger)^81^ was used to add capping groups to protein chain termini. Protonation states of all titratable residues except for E148 (Glu_ex_), E203 (Glu_in_) and E113 were assigned at pH 4.5, which is the pH at which activity is most often measured (eg. Fig. 5). Using Dabble^82^, the prepared protein structures were inserted into a pre-equilibrated palmitoyl-oleoyl-phosphatidylcholine (POPC) bilayer, the system was solvated, and sodium and chloride ions were added to neutralize the system and to obtain a final concentration of 150 mM. The final systems comprised approximately 123,000 atoms, and system dimensions were approximately 160×120×90 Å.

### Molecular dynamics simulation and analysis protocols

We used the CHARMM36m force field for proteins, the CHARMM36 force field for lipids and ions, and the TIP3P model for waters^83–85^. All simulations were performed using the Compute Unified Device Architecture (CUDA) version of particle-mesh Ewald molecular dynamics (PMEMD) in AMBER20 on graphics processing units (GPUs).

Systems were first minimized using three rounds of minimization, each consisting of 500 cycles of steepest descent followed by 500 cycles of conjugate gradient optimization. 10.0 and 5.0 kcal∙mol^−1^∙Å^−2^ harmonic restraints were applied to the protein and lipids for the first and second rounds of minimization, respectively. 1 kcal∙mol^−1^∙Å^−2^ harmonic restraints were applied to the protein for the third round of minimization. Systems were then heated from 0 K to 100 K in the NVT ensemble over 12.5 ps and then from 100 K to 310 K in the NPT ensemble over 125 ps, using 10.0 kcal∙mol^−1^∙Å^−2^ harmonic restraints applied to protein heavy atoms. Subsequently, systems were equilibrated at 310 K and 1 bar in the NPT ensemble, with harmonic restraints on the protein non-hydrogen atoms tapered off by 1.0 kcal∙mol^−1^∙Å^−2^ starting at 5.0 kcal∙mol^−1^∙Å^−2^ in a stepwise fashion every 2 ns for 10 ns, and then by 0.1 kcal∙mol^−1^∙Å^−2^ every 2 ns for 20 ns. Production simulations were performed without restraints at 310 K and 1 bar in the NPT ensemble using the Langevin thermostat and the Monte Carlo barostat, and using a timestep of 4.0 fs with hydrogen mass repartitioning^86^. Bond lengths were constrained using the SHAKE algorithm^87^. Non-bonded interactions were cut off at 9.0 Å, and long-range electrostatic interactions were calculated using the particle-mesh Ewald (PME) method with an Ewald coefficient of approximately 0.31 Å^−1^, and 4th order B-splines. The PME grid size was chosen such that the width of a grid cell was approximately 1 Å. Trajectory frames were saved every 200 ps during the production simulations. The AmberTools17 CPPTRAJ package was used to reimage trajectories^88^. Simulations were visualized and analyzed using Visual Molecular Dynamics (VMD)^89^ and PyMOL^90^.

### Isothermal Titration Calorimetry

CLC-ec1 WT and K131A protein purification was carried out as described above for WT CLC-ec1 at pH 7.5 in DM, with the following differences. First, to obtain quantities CLC ec-1 needed for ITC experiments (2 mL at ~15 μM), we used cells from 2–3 L of pellets. Second, during the final SEC step, we eluted the protein with Cl^‒^-free buffer (buffer A: 10 mM HEPES, 150 mM Na-isethionate, 5 mM anagrade DM, pH 7.5). The protein was then dialyzed at 4°C against the same buffer (250 mL) with buffer exchange every three hours for a total of four exchanges to remove trace Cl^‒^. Following dialysis, 2 mL of the protein sample was added to the sample chamber of the VP-ITC microcalorimeter (Malverne Panalytical, Malvern, United Kingdom). Titrant (30 mM KCl in buffer A) was injected into the sample chamber 10 μL at a time, once per minute for a total 180 minutes. Reference data were obtained by titrating buffer A into the protein-containing solution. The data were analyzed using MicroCal ITC-ORIGIN Analysis Software, with fitting using the ‘one set of sites’ model (keeping n = 1).

### Reconstitution and transport assays

WT and mutant CLC-ec1 were reconstituted into liposomes as described elsewhere^24,59,91^. In brief, 25 mg/ml E.coli polar lipid extract (Avanti Polar Lipids, Alabaster, Al, USA) were evaporated under vacuum, washed with pentane, and then resuspended in reconstitution buffer (300 mM KCl, 40 mM citric acid, pH = 4.3 with NaOH and 21.5 mg/mL CHAPS (3-[(3-Cholamidopropyl)dimethylammonio]-1-propanesulfonate, Biotium, Freemont, CA, USA)) to a final concentration of 20 mg/ml. CLC-ec1 protein (1–2 mg/mL in DM) was then added to achieve a protein:lipid ratio of 0.4 μg/mg and a lipid concentration between 19.5 and 19.8 mg/ml. Proteoliposomes were then aliquoted into 200-μL reconstitution samples and dialyzed over two days with 4 × 1 L reconstitution buffer. Proteoliposomes were subject to four freeze-thaw cycles and then used directly for transport assays or stored at −80°C until measured.

Flux assays were performed as described^24^. Briefly, 60 μL of reconstituted sample was buffer-exchanged using a Sephadex spin column, then diluted into 600 μL pH-4.5 flux assay buffer (300 mM K-isethionate, 2 mM citric acid and 50 μM KCl, pH 4.5). Each 200-μL reconstitution sample was used for a maximum of three flux-assay measurements (60 μL each) and the results averaged to yield a single data point (see Source Data). For each flux-assay experimental condition, reconstitutions from at least two independent protein preparations were used. Chloride and proton transport were measured in parallel using Ag/AgCl electrodes and a micro pH electrode. Data were acquired using AxosScope 9.0 (Axon Instruments, San Jose, CA, USA) and processed using Clampfit 9.0 (Axon Instruments) and Python routines. Data and transport rates shown in the manuscript were visualized in SigmaPlot 14 (Systat Software Inc., San Jose, CA, USA).

## Extended Data

**Extended Data Figure 8:**
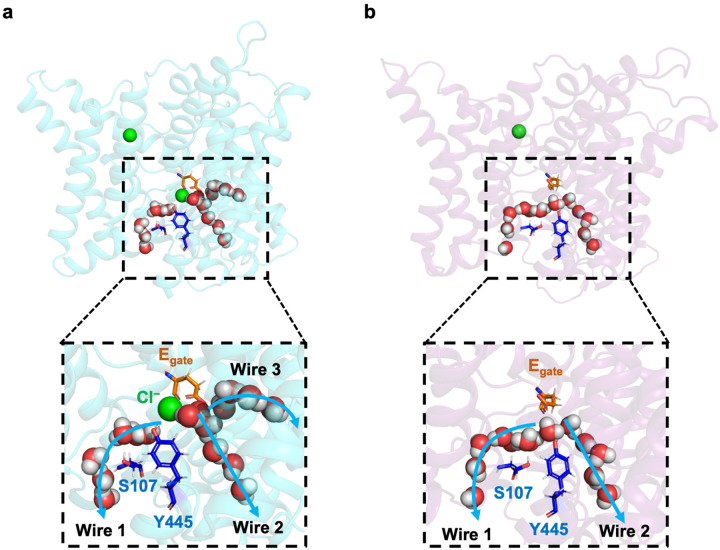
Water wires occur in the presence and absence of Cl^‒^. (a) Example snapshot of water wires formed in the presence of Cl^‒^ in the permeation pathway. (b) Example snapshot of water wires formed in the absence of Cl^‒^ in the permeation pathway

**Extended Data Figure 9.**
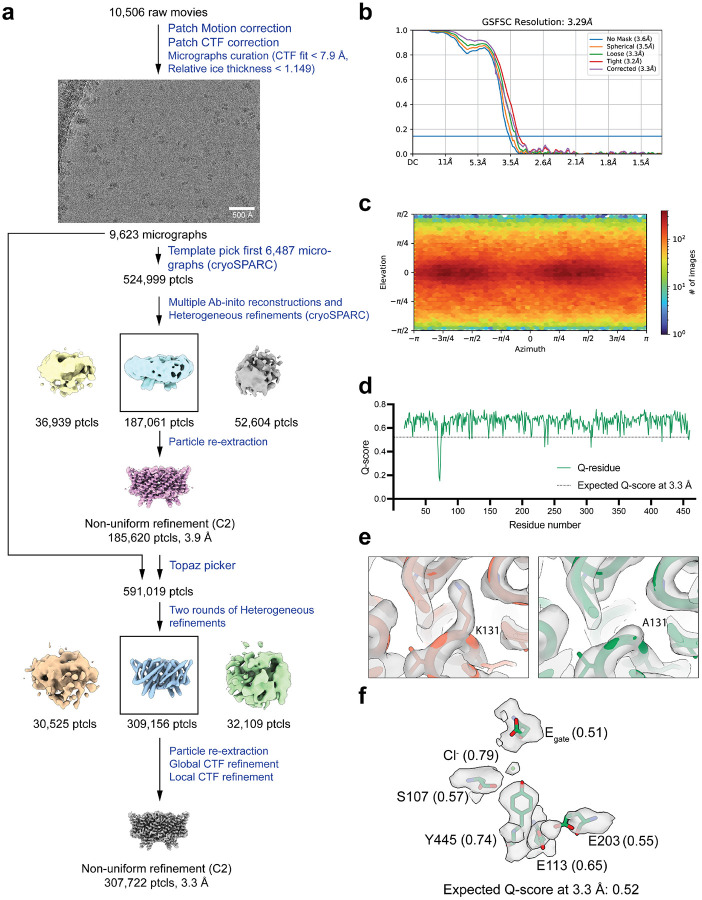
Cryo EM validation data for K131A CLC-ec1. (**a**) Cryo-EM data processing workflow. (**b**) Gold-standard FSC curve. The resolution is estimated based on FSC at 0.143. (**c**) Angular distribution plot (**d**) Per-residue Q-score as a function of residue number. The expected Q-score at the map resolution is indicated by dotted line. (**e**) The cryo-EM density and molecular model overlay in the region of K131 (left panel) and A131 (right panel). (**f**) The cryo-EM density and molecular model overlay for central Cl^‒^ binding site. The key residues and bound Cl^‒^ and their corresponding Q-score are annotated.

## Figures and Tables

**Video 1:**
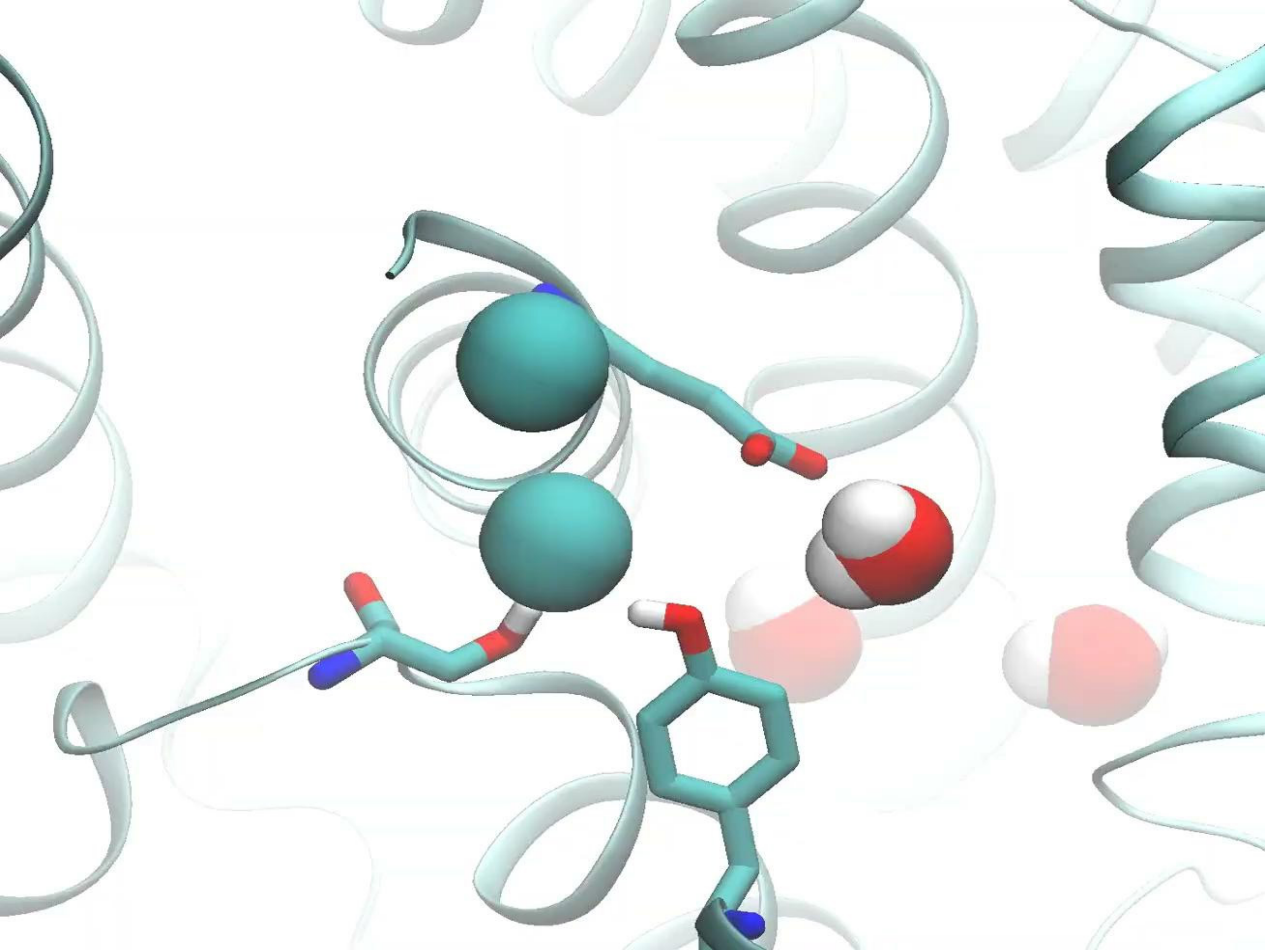
Deprotonated E_gate_ expels Cl^‒^ to the outside. A close-up view of the Cl^‒^ pathway, as seen from the membrane perspective. Inner-gate residues S017 and Y445, along with outer-gate residue E_gate_, are depicted as stick models. Cl^‒^ ions are represented as teal spheres. As E_gate_ moves into the Cl^‒^ pathway, concomitant opening of the inner gate positions the transporter to allow Cl^‒^ entry from the intracellular side.

**Table 1:** Cryo-EM data collection, refinement and validation statistics

	CLC-ec1 pH 7.5	CLC-ec1 pH 4	CLC-ec1 pH 3	CLC-ec1 K131A pH 7.5
**Data collection and processing**				
Magnification	96,000	105,000	165,000	130,000
Voltage (kV)	300	300	300	300
Electron exposure (e–/Å^2^)	80	60	60	60
Defocus range (μm)	−0.8 to −2	−0.8 to −2	−0.8 to −2	−0.8 to −2
Pixel size (Å)	0.82	0.8642	0.741	0.68
Symmetry imposed	C2	C2	C2	C2
Initial particle images (no.)	971,482	871,809	1,556,961	591,019
Final particle images (no.)	72,103	261,507	66,046	307,722
Map resolution (Å)	3.2	3.2	3.1	3.3
FSC threshold	0.143	0.143	0.143	0.143
Map resolution range (Å)	2 to 7.9	2.1 to 9.5	1.8 to 7.2	2.1 to 7.8
**Refinement**				
Initial model used (PDB code)	1OTS	1OTS	1OTS	1OTS
Model resolution (Å)	3.1	3.1	3.1	3.3
FSC threshold	0.143	0.143	0.143	0.143
Map sharpening *B* factor (Å^2^)	−147.2	−115.9		
Model composition				
Non-hydrogen	6668	6670	6486	6660
atoms	888	888	870	888
Protein residues	2	2	4	2
Ligands				
*B* factors (Å^2^)				
Protein	50.12	49.39	59.92	43.67
Ligand	70.43	59.92	86.14	88.32
R.m.s. deviations				
Bond lengths (Å)	0.003	0.002	0.002	0.003
Bond angles (°)	0.511	0.535	0.529	0.541
**Validation**				
MolProbity score	1.01	0.97	1.22	1.07
Clashscore	2.35	1.98	3.17	1.98
Poor rotamers (%)	0.30	0.60	0.62	0.60
Ramachandran plot				
Favored (%)	98.42	97.96	97.46	97.51
Allowed (%)	1.58	2.04	2.54	2.49
Disallowed (%)	0.00	0.00	0.00	0.00

**Table 2:** Turnover rates and stoichiometry of the proteins in this study. Transport rates and stoichiometry are displayed as the mean ± SEM. The number of flux assays for each experiment is shown by n and stem from at least two independent protein preparations. The stoichiometry is calculated from the ratio of the CI^‒^ and H^+^ transport rates.

CLC-ec1	Transport rate (s^−1^)		Cl^−^/H^+^ stoichiometry	n
	Cl^−^	H^+^		
**WT**	2300 ± 100	1100 ± 40	2.0 ± 0.1	6
**K131A**	110 ± 30	40 ± 10	3.0 ± 0.5	4
**K131M**	130 ± 30	40 ± 10	3.4 ± 0.5	3
**E148A**	470 ± 40	n/a	n/a	5
**K131A/E148A**	340 ± 30	n/a	n/a	4
**K131M/E148A**	460 ± 150	n/a	n/a	3

## Data Availability

The mass spectrometry proteomics data have been deposited to the ProteomeXchange Consortium via the PRIDE [1] partner repository with the dataset identifier PXD058693” where the reference is PubMed ID: 34723319. The DNA constructs used in this study are available from M.M. upon request. Models and associated cryo-EM maps have been deposited into the Protein Data Bank (PDB) and the Electron Microscopy Data Bank (EMDB) under the following accession codes: CLC-ec1 pH 7.5 (PDB ID 9095; EMD-70242); CLC-ec1 pH 4.0 (PDB ID 9096; EMD-70243); CLC-ec1 pH 3.0 (PDB ID 9097; EMD-70244); CLC-ec1 K131A pH 7.5 (PDB ID 9098; EMD-70245). The MD simulation data that support this study are available from R.O.D. upon request.

## References

[1] Accardi A. & Miller C. Secondary active transport mediated by a prokaryotic homologue of ClC Cl-channels. Nature 427, 803–7 (2004). 14985752 10.1038/nature02314

[2] Miller C. ClC chloride channels viewed through a transporter lens. Nature 440, 484–9 (2006). 16554809 10.1038/nature04713

[3] Lisal J. & Maduke M. The ClC-0 chloride channel is a ‘broken’ Cl-/H+ antiporter. Nat Struct Mol Biol 15, 805–10 (2008). 18641661 10.1038/nsmb.1466 PMC2559860

[4] Jentsch T.J. & Pusch M. CLC Chloride Channels and Transporters: Structure, Function, Physiology, and Disease. Physiol Rev 98, 1493–1590 (2018). 29845874 10.1152/physrev.00047.2017

[5] Koster A.K. & Maduke M. CLC Chloride Channels and Transporters. in Textbook of Ion Channels Vol. II Properties, Function, and Pharmacology of the Superfamilies (eds. Zheng J. & Trudeau M.C.) 193–208 (CRC Press, Boca Raton, FL, 2023).

[6] Bose S., He H. & Stauber T. Neurodegeneration Upon Dysfunction of Endosomal/Lysosomal CLC Chloride Transporters. Front Cell Dev Biol 9, 639231 (2021). 33708769 10.3389/fcell.2021.639231 PMC7940362

[7] Duncan A.R. Unique variants in CLCN3, encoding an endosomal anion/proton exchanger, underlie a spectrum of neurodevelopmental disorders. Am J Hum Genet 108, 1450–1465 (2021). 34186028 10.1016/j.ajhg.2021.06.003 PMC8387284

[8] Zifarelli G. The Role of the Lysosomal Cl(−)/H(+) Antiporter ClC-7 in Osteopetrosis and Neurodegeneration. Cells 11(2022). 10.3390/cells11030366 PMC8833911 35159175

[9] Coppola M.A. Biophysical Aspects of Neurodegenerative and Neurodevelopmental Disorders Involving Endo-/Lysosomal CLC Cl(−)/H(+) Antiporters. Life (Basel) 13(2023). 10.3390/life13061317 PMC10303033 37374100

[10] Palmer E.E. Functional and clinical studies reveal pathophysiological complexity of CLCN4-related neurodevelopmental condition. Mol Psychiatry 28, 668–697 (2023). 36385166 10.1038/s41380-022-01852-9 PMC9908558

[11] Dieguez L. Dent’s Disease: A Cause of Monogenic Kidney Stones and Nephrocalcinosis. J Pers Med 14(2024). 10.3390/jpm14060623 PMC11204629 38929844

[12] Iyer H. & Talbot W.S. The Cl- transporter ClC-7 is essential for phagocytic clearance by microglia. J Cell Sci 137(2024). 10.1242/jcs.261616 PMC10911276 38294065

[13] McIlwain B.C., Ruprecht M.T. & Stockbridge R.B. Membrane Exporters of Fluoride Ion. Annu Rev Biochem 90, 559–579 (2021). 33492991 10.1146/annurev-biochem-071520-112507 PMC8217344

[14] Liu L., Li X., Wang C., Ni Y. & Liu X. The Role of Chloride Channels in Plant Responses to NaCl. Int J Mol Sci 25(2023). 10.3390/ijms25010019 PMC10778697 38203189

[15] Iyer R., Iverson T.M., Accardi A. & Miller C. A biological role for prokaryotic ClC chloride channels. Nature 419, 715–8 (2002). 12384697 10.1038/nature01000

[16] Kim M., Choi N., Choi E. & Lee E.J. ClC Chloride Channels in Gram-Negative Bacteria and Its Role in the Acid Resistance Systems. J Microbiol Biotechnol 33, 857–863 (2023). 37100762 10.4014/jmb.2303.03009 PMC10394335

[17] Maduke M., Pheasant D.J. & Miller C. High-level expression, functional reconstitution, and quaternary structure of a prokaryotic ClC-type chloride channel. J Gen Physiol 114, 713–22 (1999). 10539975 10.1085/jgp.114.5.713 PMC2230540

[18] Mindell J.A., Maduke M., Miller C. & Grigorieff N. Projection structure of a ClC-type chloride channel at 6.5 A resolution. Nature 409, 219–23 (2001). 11196649 10.1038/35051631

[19] Dutzler R., Campbell E.B., Cadene M., Chait B.T. & MacKinnon R. X-ray structure of a ClC chloride channel at 3.0 A reveals the molecular basis of anion selectivity. Nature 415, 287–94 (2002). 11796999 10.1038/415287a

[20] Dutzler R. A structural perspective on ClC channel and transporter function. FEBS Lett 581, 2839–44 (2007). 17452037 10.1016/j.febslet.2007.04.016

[21] Accardi A. Structure and gating of CLC channels and exchangers. J Physiol 593, 4129–38 (2015). 26148215 10.1113/JP270575 PMC4594288

[22] Picollo A. Vesicular CLC chloride/proton exchangers in health and diseases. Front Pharmacol 14, 1295068 (2023). 38027030 10.3389/fphar.2023.1295068 PMC10662042

[23] Dutzler R., Campbell E.B. & MacKinnon R. Gating the selectivity filter in ClC chloride channels. Science 300, 108–12 (2003). 12649487 10.1126/science.1082708

[24] Chavan T.S. A CLC-ec1 mutant reveals global conformational change and suggests a unifying mechanism for the CLC Cl(−)/H(+) transport cycle. Elife 9(2020). 10.7554/eLife.53479 PMC7253180 32310757

[25] Fortea E. Structural basis of pH-dependent activation in a CLC transporter. Nat Struct Mol Biol 31, 644–656 (2024). 38279055 10.1038/s41594-023-01210-5 PMC11262703

[26] Picollo A., Malvezzi M., Houtman J.C. & Accardi A. Basis of substrate binding and conservation of selectivity in the CLC family of channels and transporters. Nat Struct Mol Biol 16, 1294–301 (2009). 19898476 10.1038/nsmb.1704 PMC2920496

[27] Picollo A., Xu Y., Johner N., Berneche S. & Accardi A. Synergistic substrate binding determines the stoichiometry of transport of a prokaryotic H(+)/Cl(−) exchanger. Nat Struct Mol Biol 19, 525–31, S1 (2012). 22484316 10.1038/nsmb.2277 PMC3348462

[28] Nguitragool W. & Miller C. CLC Cl /H+ transporters constrained by covalent cross-linking. Proc Natl Acad Sci U S A 104, 20659–65 (2007). 18093952 10.1073/pnas.0708639104 PMC2409211

[29] Chadda R. & Robertson J.L. Measuring Membrane Protein Dimerization Equilibrium in Lipid Bilayers by Single-Molecule Fluorescence Microscopy. Methods Enzymol 581, 53–82 (2016). 27793292 10.1016/bs.mie.2016.08.025 PMC5568537

[30] Khantwal C.M. Revealing an outward-facing open conformational state in a CLC Cl(−)/H(+) exchange transporter. Elife 5(2016). 10.7554/eLife.11189 PMC4769167 26799336

[31] Accardi A. Separate ion pathways in a Cl−/H+ exchanger. J Gen Physiol 126, 563–70 (2005). 16316975 10.1085/jgp.200509417 PMC2266597

[32] Han W., Cheng R.C., Maduke M.C. & Tajkhorshid E. Water access points and hydration pathways in CLC H+/Cl− transporters. Proc Natl Acad Sci U S A 111, 1819–24 (2014). 24379362 10.1073/pnas.1317890111 PMC3918786

[33] Kuang Z., Mahankali U. & Beck T.L. Proton pathways and H+/Cl− stoichiometry in bacterial chloride transporters. Proteins 68, 26–33 (2007). 17410581 10.1002/prot.21441

[34] Wang D. & Voth G.A. Proton transport pathway in the ClC Cl−/H+ antiporter. Biophys J 97, 121–31 (2009). 19580750 10.1016/j.bpj.2009.04.038 PMC2711357

[35] Ko Y.J. & Jo W.H. Secondary water pore formation for proton transport in a ClC exchanger revealed by an atomistic molecular-dynamics simulation. Biophysical Journal 98, 2163–9 (2010). 20483324 10.1016/j.bpj.2010.01.043 PMC2872259

[36] Kieseritzky G. & Knapp E.W. Charge transport in the ClC-type chloride-proton anti-porter from Escherichia coli. Journal of Biological Chemistry 286, 2976–86 (2011). 21059656 10.1074/jbc.M110.163246 PMC3024792

[37] Jiang T., Han W., Maduke M. & Tajkhorshid E. Molecular Basis for Differential Anion Binding and Proton Coupling in the Cl(−)/H(+) Exchanger ClC-ec1. J Am Chem Soc 138, 3066–75 (2016). 26880377 10.1021/jacs.5b12062 PMC4898471

[38] Leisle L. Divergent Cl(−) and H(+) pathways underlie transport coupling and gating in CLC exchangers and channels. Elife 9(2020). 10.7554/eLife.51224 PMC7274781 32343228

[39] Wang Z., Swanson J.M.J. & Voth G.A. Local conformational dynamics regulating transport properties of a Cl(−) /H(+) antiporter. J Comput Chem 41, 513–519 (2020). 31633205 10.1002/jcc.26093 PMC7184886

[40] Accardi A., Lobet S., Williams C., Miller C. & Dutzler R. Synergism between halide binding and proton transport in a CLC-type exchanger. J Mol Biol 362, 691–9 (2006). 16949616 10.1016/j.jmb.2006.07.081

[41] Nguitragool W. & Miller C. Uncoupling of a CLC Cl−/H+ exchange transporter by polyatomic anions. J Mol Biol 362, 682–90 (2006). 16905147 10.1016/j.jmb.2006.07.006

[42] Miller C. Q-cubed mutant cues clues to CLC antiport mechanism. J Gen Physiol 153(2021). 10.1085/jgp.202112868 PMC7894039 33600550

[43] Matulef K. & Maduke M. Side-dependent inhibition of a prokaryotic ClC by DIDS. Biophys J 89, 1721–30 (2005). 15994902 10.1529/biophysj.105.066522 PMC1366676

[44] Bosshart P.D. & Fotiadis D. Secondary Active Transporters. Subcell Biochem 92, 275–299 (2019). 31214990 10.1007/978-3-030-18768-2_9

[45] Garaeva A.A. & Slotboom D.J. Elevator-type mechanisms of membrane transport. Biochem Soc Trans 48, 1227–1241 (2020). 32369548 10.1042/BST20200290 PMC7329351

[46] Licht J.A., Berry S.P., Gutierrez M.A. & Gaudet R. They all rock: A systematic comparison of conformational movements in LeuT-fold transporters. Structure 32, 1528–1543 e3 (2024). 39025067 10.1016/j.str.2024.06.015 PMC11380583

[47] Bell S.P., Curran P.K., Choi S. & Mindell J.A. Site-directed fluorescence studies of a prokaryotic ClC antiporter. Biochemistry 45, 6773–82 (2006). 16734414 10.1021/bi0523815

[48] Elvington S.M. & Maduke M. Thinking outside the crystal: complementary approaches for examining transporter conformational change. Channels (Austin) 2, 373–9 (2008). 18989097 10.4161/chan.2.5.6903

[49] Elvington S.M., Liu C.W. & Maduke M.C. Substrate-driven conformational changes in ClC-ec1 observed by fluorine NMR. EMBO J 28, 3090–102 (2009). 19745816 10.1038/emboj.2009.259 PMC2771095

[50] Abraham S.J. 13C NMR detects conformational change in the 100-kD membrane transporter ClC-ec1. J Biomol NMR 61, 209–26 (2015). 25631353 10.1007/s10858-015-9898-7 PMC4398623

[51] Heath G.R. Localization atomic force microscopy. Nature 594, 385–390 (2021). 34135520 10.1038/s41586-021-03551-x PMC8697813

[52] Accardi A., Kolmakova-Partensky L., Williams C. & Miller C. Ionic currents mediated by a prokaryotic homologue of CLC Cl- channels. J Gen Physiol 123, 109–19 (2004). 14718478 10.1085/jgp.200308935 PMC2217429

[53] Coppieters ‘t Wallant K. & Martens C. Hydrogen-deuterium exchange coupled to mass spectrometry: A multifaceted tool to decipher the molecular mechanism of transporters. Biochimie 205, 95–101 (2023). 36037883 10.1016/j.biochi.2022.08.014

[54] Ozohanics O. & Ambrus A. Hydrogen-Deuterium Exchange Mass Spectrometry: A Novel Structural Biology Approach to Structure, Dynamics and Interactions of Proteins and Their Complexes. Life (Basel) 10(2020). 10.3390/life10110286 PMC7696067 33203161

[55] Pintilie G. Measurement of atom resolvability in cryo-EM maps with Q-scores. Nat Methods 17, 328–334 (2020). 32042190 10.1038/s41592-020-0731-1 PMC7446556

[56] Wraight C.A. Chance and design--proton transfer in water, channels and bioenergetic proteins. Biochim Biophys Acta 1757, 886–912 (2006). 16934216 10.1016/j.bbabio.2006.06.017

[57] Kratochvil H.T. Transient water wires mediate selective proton transport in designed channel proteins. Nat Chem 15, 1012–1021 (2023). 37308712 10.1038/s41557-023-01210-4 PMC10475958

[58] Faraldo-Gomez J.D. & Roux B. Electrostatics of ion stabilization in a ClC chloride channel homologue from Escherichia coli. J Mol Biol 339, 981–1000 (2004). 15165864 10.1016/j.jmb.2004.04.023

[59] Walden M. Uncoupling and turnover in a Cl-/H+ exchange transporter. J Gen Physiol 129, 317–29 (2007). 17389248 10.1085/jgp.200709756 PMC2151619

[60] Baker J.A., Wong W.C., Eisenhaber B., Warwicker J. & Eisenhaber F. Charged residues next to transmembrane regions revisited: “Positive-inside rule” is complemented by the “negative inside depletion/outside enrichment rule”. BMC Biol 15, 66 (2017). 28738801 10.1186/s12915-017-0404-4 PMC5525207

[61] Zhang X.D., Li Y., Yu W.P. & Chen T.Y. Roles of K149, G352, and H401 in the channel functions of ClC-0: testing the predictions from theoretical calculations. J Gen Physiol 127, 435–47 (2006). 16567465 10.1085/jgp.200509460 PMC2151512

[62] Engh A.M., Faraldo-Gomez J.D. & Maduke M. The role of a conserved lysine in chloride- and voltage-dependent ClC-0 fast gating. J Gen Physiol 130, 351–63 (2007). 17846165 10.1085/jgp.200709760 PMC2151651

[63] Yuan J.H. Genetic spectrum and founder effect of non-dystrophic myotonia: a Japanese case series study. J Neurol 269, 6406–6415 (2022). 35907044 10.1007/s00415-022-11305-6

[64] Brenes O., Pusch M. & Morales F. ClC-1 Chloride Channel: Inputs on the Structure-Function Relationship of Myotonia Congenita-Causing Mutations. Biomedicines 11(2023). 10.3390/biomedicines11102622 PMC10604815 37892996

[65] Leray X. Tonic inhibition of the chloride/proton antiporter ClC-7 by PI(3,5)P2 is crucial for lysosomal pH maintenance. Elife 11(2022). 10.7554/eLife.74136 PMC9242644 35670560

[66] Carpaneto A., Boccaccio A., Lagostena L., Di Zanni E. & Scholz-Starke J. The signaling lipid phosphatidylinositol-3,5-bisphosphate targets plant CLC-a anion/H(+) exchange activity. EMBO Rep 18, 1100–1107 (2017). 28536248 10.15252/embr.201643814 PMC5494527

[67] He H. Mutations in CLCN6 as a Novel Genetic Cause of Neuronal Ceroid Lipofuscinosis in Patients and a Murine Model. Ann Neurol 96, 608–624 (2024). 38877824 10.1002/ana.27002

[68] Grieschat M., Guzman R.E., Langschwager K., Fahlke C. & Alekov A.K. Metabolic energy sensing by mammalian CLC anion/proton exchangers. EMBO Rep 21, e47872 (2020). 32390228 10.15252/embr.201947872 PMC7271328

[69] He J. Cryo-EM structure of the plant nitrate transporter AtCLCa reveals characteristics of the anion-binding site and the ATP-binding pocket. J Biol Chem 299, 102833 (2023). 36581207 10.1016/j.jbc.2022.102833 PMC9898749

[70] Zhang B. Molecular basis of ClC-6 function and its impairment in human disease. Sci Adv 9, eadg4479 (2023). 37831762 10.1126/sciadv.adg4479 PMC10575590

[71] Wan Y. Structural basis of adenine nucleotides regulation and neurodegenerative pathology in ClC-3 exchanger. Nat Commun 15, 6654 (2024). 39107281 10.1038/s41467-024-50975-w PMC11303396

[72] Skerra A. Use of the tetracycline promoter for the tightly regulated production of a murine antibody fragment in E.coli. Gene 151, 131–135 (1994). 7828861 10.1016/0378-1119(94)90643-2

[73] Sambrook J., Fritsch E.F. & Maniatis T. Molecular Cloning, A Laboratory Manual, (Cold Spring Harbor Laboratory Press, Cold Spring Harbor, NY, 1989).

[74] Chien C.H. An Adaptable Phospholipid Membrane Mimetic System for Solution NMR Studies of Membrane Proteins. J Am Chem Soc 139, 14829–14832 (2017). 28990386 10.1021/jacs.7b06730 PMC6109379

[75] Yang M. Recombinant Nepenthesin II for Hydrogen/Deuterium Exchange Mass Spectrometry. Anal Chem 87, 6681–7 (2015). 25993527 10.1021/acs.analchem.5b00831

[76] Majumdar R. Minimizing carry-over in an online pepsin digestion system used for the H/D exchange mass spectrometric analysis of an IgG1 monoclonal antibody. J Am Soc Mass Spectrom 23, 2140–8 (2012). 22993047 10.1007/s13361-012-0485-9

[77] Trcka F. Human Stress-inducible Hsp70 Has a High Propensity to Form ATP-dependent Antiparallel Dimers That Are Differentially Regulated by Cochaperone Binding. Mol Cell Proteomics 18, 320–337 (2019). 30459217 10.1074/mcp.RA118.001044 PMC6356074

[78] Kavan D.a.M.., P. MSTools—Web based application for visualization and presentation of HXMS data. International Journal of Mass Spectrometry 302, 53–58 (2011).

[79] Pettersen E.F. UCSF ChimeraX: Structure visualization for researchers, educators, and developers. Protein Sci 30, 70–82 (2021). 32881101 10.1002/pro.3943 PMC7737788

[80] Lomize M.A., Lomize A.L., Pogozheva I.D. & Mosberg H.I. OPM: orientations of proteins in membranes database. Bioinformatics 22, 623–5 (2006). 16397007 10.1093/bioinformatics/btk023

[81] Jacobson M.P., Friesner R.A., Xiang Z. & Honig B. On the role of the crystal environment in determining protein side-chain conformations. J Mol Biol 320, 597–608 (2002). 12096912 10.1016/s0022-2836(02)00470-9

[82] Betz R. Dabble. (2017).

[83] Huang J. CHARMM36m: an improved force field for folded and intrinsically disordered proteins. Nat Methods 14, 71–73 (2017). 27819658 10.1038/nmeth.4067 PMC5199616

[84] Klauda J.B. Update of the CHARMM all-atom additive force field for lipids: validation on six lipid types. J Phys Chem B 114, 7830–43 (2010). 20496934 10.1021/jp101759q PMC2922408

[85] Beglov D. & Roux B. Finite representation of an infinite bulk system: Solvent boundary potential for computer simulations. J. Chem. Phys. 100, 9050–9063 (1994).

[86] Hopkins C.W., Le Grand S., Walker R.C. & Roitberg A.E. Long-Time-Step Molecular Dynamics through Hydrogen Mass Repartitioning. J Chem Theory Comput 11, 1864–74 (2015). 26574392 10.1021/ct5010406

[87] Ryckaert J.-P., Ciccotti G., & Berendsen H. J. C. . Numerical Integration of the Cartesian Equations of Motion of a System with Constraints: Molecular Dynamics of n-Alkanes. Journal of Computational Physics 23, 327–341 (1977).

[88] Roe D.R. & Cheatham T.E. 3rd. PTRAJ and CPPTRAJ: Software for Processing and Analysis of Molecular Dynamics Trajectory Data. J Chem Theory Comput 9, 3084–95 (2013). 26583988 10.1021/ct400341p

[89] Humphrey W., Dalke A. & Schulten K. VMD: visual molecular dynamics. J Mol Graph 14, 33–8, 27–8 (1996). 8744570 10.1016/0263-7855(96)00018-5

[90] The PyMOL Molecular Graphics System, Version 2.0 Schrödinger, LLC.

[91] Lim H.H., Shane T. & Miller C. Intracellular proton access in a Cl(−)/H(+) antiporter. PLoS Biol 10, e1001441 (2012). 23239938 10.1371/journal.pbio.1001441 PMC3519907

[92] Schrecker M., Korobenko J. & Hite R.K. Cryo-EM structure of the lysosomal chloride-proton exchanger CLC-7 in complex with OSTM1. Elife 9(2020). 10.7554/eLife.59555 PMC7440919 32749217

[93] Park E. & MacKinnon R. Structure of the CLC-1 chloride channel from Homo sapiens. Elife 7(2018). 10.7554/eLife.36629 PMC6019066 29809153

[94] Feng L., Campbell E.B., Hsiung Y. & MacKinnon R. Structure of a eukaryotic CLC transporter defines an intermediate state in the transport cycle. Science 330, 635–41 (2010). 20929736 10.1126/science.1195230 PMC3079386

[95] Xu M. CryoEM structures of the human CLC-2 voltage-gated chloride channel reveal a ball-andchain gating mechanism. Elife 12(2024). 10.7554/eLife.90648 PMC10942593 38345841

[96] Pintilie G. & Chiu W. Assessment of structural features in Cryo-EM density maps using SSE and side chain Z-scores. J Struct Biol 204, 564–571 (2018). 30144506 10.1016/j.jsb.2018.08.015 PMC6525962

